# Predicting Network Activity from High Throughput Metabolomics

**DOI:** 10.1371/journal.pcbi.1003123

**Published:** 2013-07-04

**Authors:** Shuzhao Li, Youngja Park, Sai Duraisingham, Frederick H. Strobel, Nooruddin Khan, Quinlyn A. Soltow, Dean P. Jones, Bali Pulendran

**Affiliations:** 1 Emory Vaccine Center, Emory University, Atlanta, Georgia, United States of America; 2 Yerkes National Primate Research Center, Emory University, Atlanta, Georgia, United States of America; 3 Department of Medicine, Emory University, Atlanta, Georgia, United States of America; 4 College of Pharmacy, Korea University, Seoul, South Korea; 5 Mass Spectrometry Center, Emory University, Atlanta, Georgia, United States of America; The Centre for Research and Technology, Hellas, Greece

## Abstract

The functional interpretation of high throughput metabolomics by mass spectrometry is hindered by the identification of metabolites, a tedious and challenging task. We present a set of computational algorithms which, by leveraging the collective power of metabolic pathways and networks, predict functional activity directly from spectral feature tables without a priori identification of metabolites. The algorithms were experimentally validated on the activation of innate immune cells.

## Author Summary

Mass spectrometry based untargeted metabolomics can now profile several thousand of metabolites simultaneously. However, these metabolites have to be identified before any biological meaning can be drawn from the data. Metabolite identification is a challenging and low throughput process, therefore becomes the bottleneck of the filed. We report here a novel approach to predict biological activity directly from mass spectrometry data without a priori identification of metabolites. By unifying network analysis and metabolite prediction under the same computational framework, the organization of metabolic networks and pathways helps resolve the ambiguity in metabolite prediction to a large extent. We validated our algorithms on a set of activation experiment of innate immune cells. The predicted activities were confirmed by both gene expression and metabolite identification. This method shall greatly accelerate the application of high throughput metabolomics, as the tedious task of identifying hundreds of metabolites upfront can be shifted to a handful of validation experiments after our computational prediction.

## Introduction

Knowledge of many metabolic pathways has accumulated over the past century. For instance, glycolysis, citric acid cycle and oxidative phosphorylation fuel cellular processes through the generation of adenosine triphosphate; glycans and cholesterols not only serve as structural blocks but also mediate intercellular communication. In fact, metabolites pervade every aspect of life [1], [2]. Their roles are increasingly appreciated, as advancing tools allow deeper scientific investigations. The most notable progresses in recent years come from metabolomics and genome-scale metabolic models.

Metabolomics is the emerging field of comprehensive profiling of metabolites. As metabolites are the direct readout of functional activity, metabolomics fills in a critical gap in the realm of systems biology, complementing genomics and proteomics [3]–[6]. The technical platforms of metabolomics are mainly based on mass spectromety and nuclear magnetic resonance [4], [7]. Among them, untargeted LC/MS (liquid chromatography coupled mass spectrometry), especially on high resolution spectrometers, produces unparalleled throughput, measuring thousands of metabolite features simultaneously [5], [8]–[10].

On the other hand, genome-scale metabolic models have been largely driven by genomics, as the total list of metabolic enzymes of a species can be derived from its genome sequence [11], [12]. The reconstruction of microbial metabolic network models is an established process [13], [14]. Intense manual curation, however, was required in the building of two high-quality human models [15], [16], which were followed by a number of derivatives [17]–[20]. The coverage of these metabolic models greatly exceeds the conventional pathways.

Even though they are a perfect match in theory, metabolomics and genome-scale metabolic models have had little overlap so far. The use of metabolomics in building metabolic models is rare [21], due to the scarcity of well annotated metabolomics data. The application of genome-scale metabolic models to metabolomics data is not common either [22]. The limited throughput of targeted metabolomics usually does not motivate large scale network analysis. Untargeted metabolomics cannot move onto pathway and network analysis without knowing the identity of metabolites.

**Figure 1:**
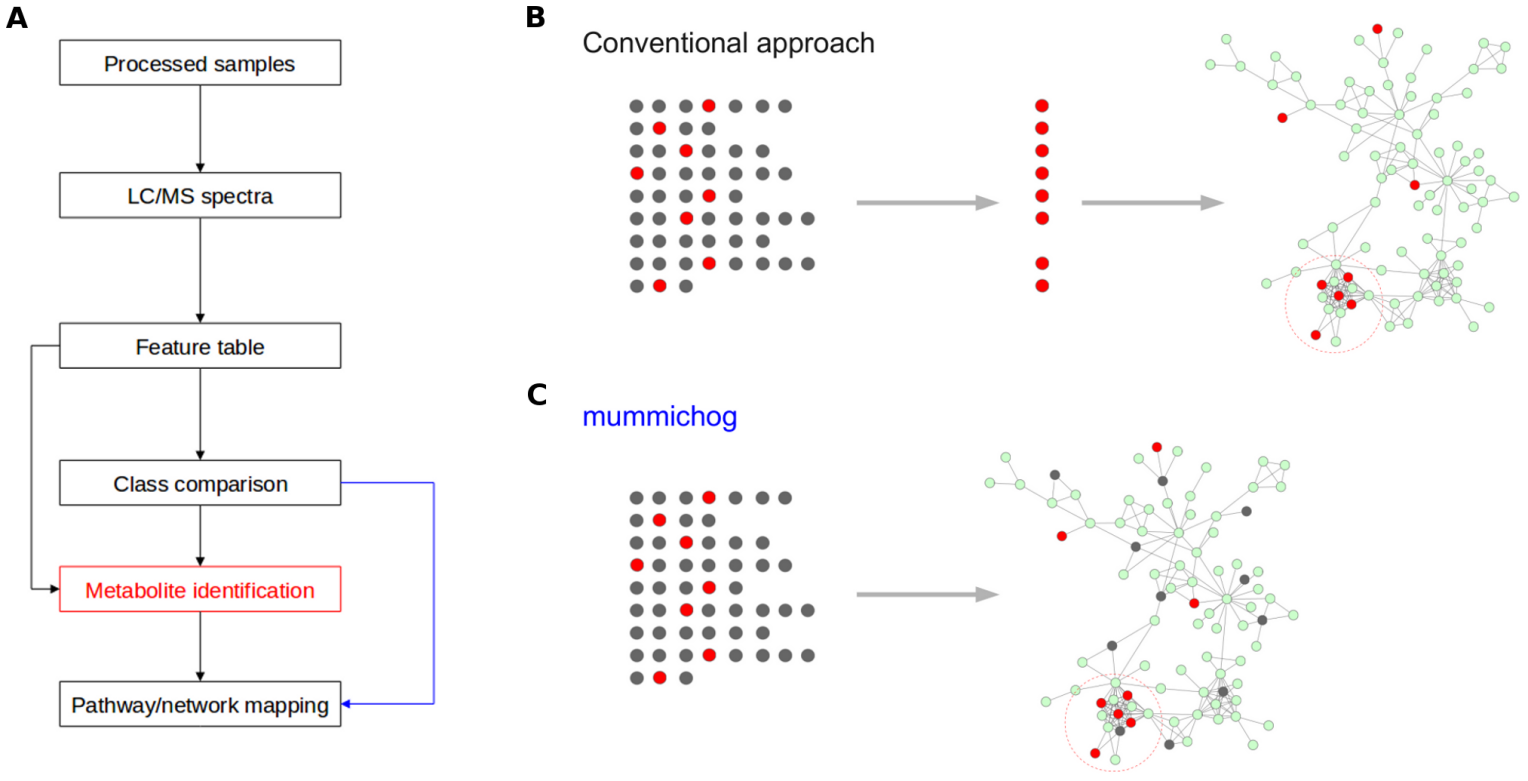
Mummichog redefines the work flow of untargeted metabolomics. A) In the work flow of untargeted metabolomics, the conventional approach requires the metabolites to be identified before pathway/network analysis, while mummichog (blue arrow) predicts functional activity bypassing metabolite identification. B) Each row of dots represent possible matches of metabolites from one *m/z* feature, red the true metabolite, gray the false matches. The conventional approach first requires the identification of metabolites before mapping them to the metabolic network. C) *mummichog* maps all possible metabolite matches to the network and looks for local enrichment, which reflects the true activity because the false matches will distribute randomly.

A typical work flow of untargeted metabolomics is illustrated in Figure 1A. After ionized molecules are scanned in the spectrometer, the spectral peaks are extracted, quantified and aligned into a feature table. At this point, each feature is identified by a mass-to-charge ratio (*m/z*) and retention time in chromatography, but its chemical identity is not known. To assign a spectral feature to a *bona fide* metabolite, it usually involves tandem mass spectrometry to examine the fragmentation pattern of a specific feature, or coelution of isotopically labeled known references - both are inherently low throughput. Considerable effort is needed to build a spectral library, which is often of limited size and interoperability. Thus, metabolite identification forms the bottleneck of untargeted metabolomics [23].

A number of informatics tools have been developed for LC/MS metabolomics, ranging from feature extraction [24]–[26], pathway analysis and visualization [27]–[29] to work flow automation [30]–[32]. Yet, whereas pathway and network analysis is concerned, the existing tools require identified metabolites to start with. Computational prediction of metabolite identity, based on *m/z* alone, is deemed inadequate as a single *m/z* feature can match multiple metabolites even with high instrumental accuracy [33], [34], and multiple forms of the same metabolite often exist in the mass spectra [35]. Although automated MS/MS (tandem mass spectrometry) search in databases is improving the efficiency of metabolite identification [36], [37], this requires additional targeted experiments and relies on extensive databases, where data from different platforms often do not match. How to bring untargeted metabolomics data to biological interpretation remains a great challenge.

In this paper, we report a novel approach of predicting network activity from untargeted metabolomics without upfront identification of metabolites, thus greatly accelerating the work flow. This is possible because the collective power in metabolic networks helps resolve the ambiguity in metabolite prediction. We will describe the computational algorithms, and demonstrate their application to the activation of innate immune cells.

## Results

Full MS scan in an untargeted metabolomics experiment gives the most power of high throughput profiling, producing several thousand of features routinely. Our goal is to predict biological activity in a network of metabolites directly from the feature table, bypassing the bottleneck of metabolite identification (Figure 1A). As illustrated in Figure 1B, each spectral feature can match to several metabolite candidates, albeit the true identity is not known. The conventional work flow is to identify each metabolite experimentally before mapping them onto metabolic pathways/networks. Instead of treating metabolite identification and metabolic pathway/network analysis as two separate steps, we argue that there is collective power in the organization of metabolic networks, which can be leveraged when the two steps are unified under one theoretical framework. In other words, if a list of significant spectral features reflects a biological activity, the true metabolites they represent should show enrichment on a local structure in the metabolic network [22], while the falsely matched metabolites are distributed more randomly. This gives us a means to predict functional activities without upfront identification of metabolites (Figure 1C).

The software implementation of our approach is named *mummichog* (*mummichog* is an American Indian term for by groups, also name of a small fish which live by groups). From user input, *mummichog* requires two lists of *m/z* features, the significant list 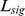 (e.g. selected by univariate statistics) and the reference list 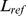 (all features detected in the MS experiment). From the *m/z* features in 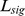, *mummichog* computes all possibly matched metabolites, and searches the reference metabolic network for all the modules that can be formed by these tentative metabolites. Random lists of *m/z* features are drawn from 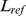 many times to estimate the null distribution of module activities. The statistical significance of modules from 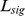 can then be calculated on this null distribution. In return, the predicted metabolites in these significant modules (and similarly for pathways) are more likely to be true, and they form the “activity network” for this particular experiment.

Details of module analysis, pathway analysis and activity network are given below, after a treatment of the reference metabolic network model.

### The reference metabolic network model

**Figure 2:**
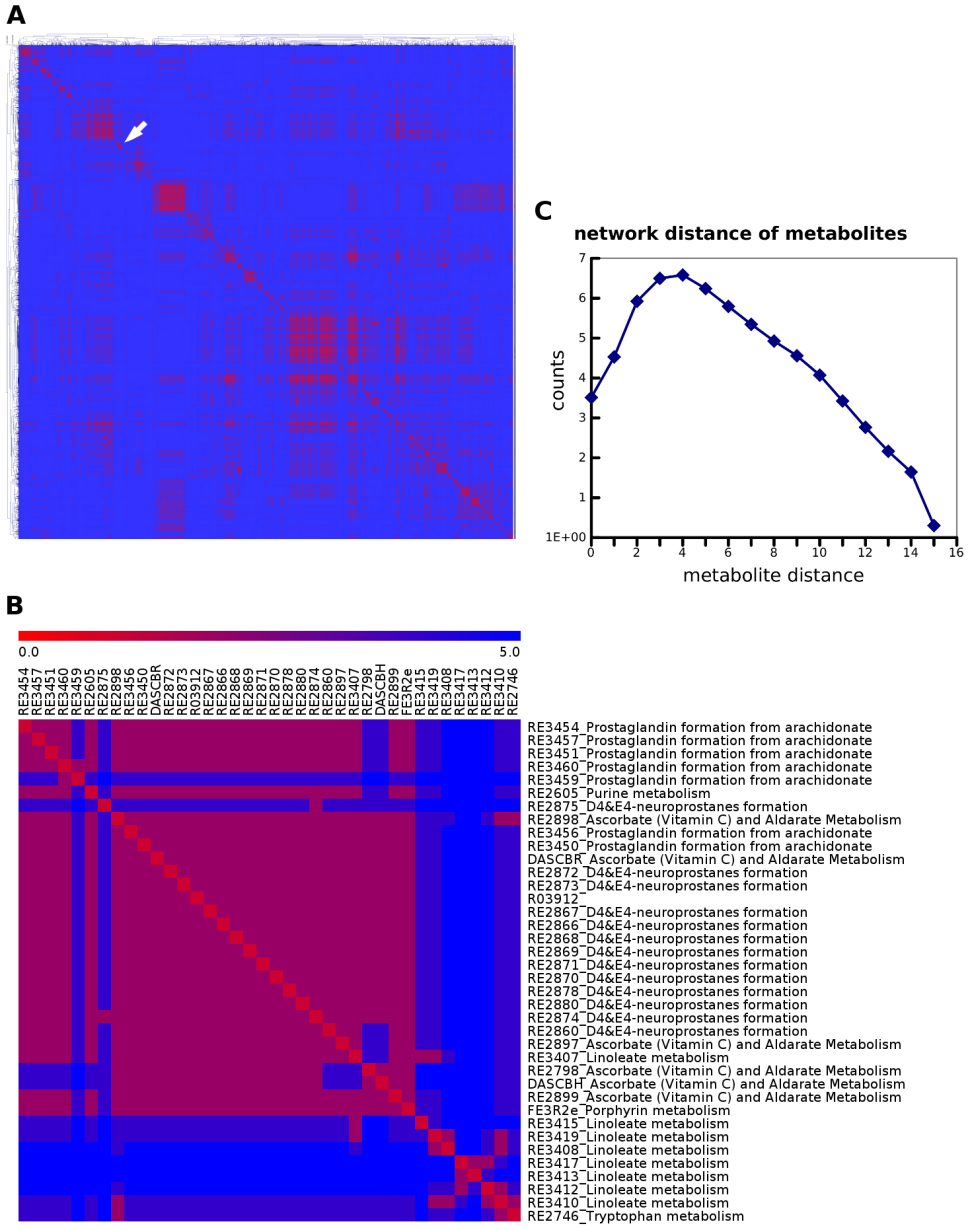
Modular organization of human metabolic network. A) Hierarchical clustering of the network by the steps between 4204 metabolic reactions, where the warmer color codes for fewer steps. Each red island represents a cluster of closely connected reactions. B) An insert by the while arrow in A. This demonstrates that network modules and pathways correlate with but not equate to each other. C) When measured by reaction steps between metabolites, most metabolites are connected in no more than four steps. This serves as a practical guide in searching subnetworks in the total metabolic network.

The genome-scale human metabolic network in *mummichog* is based on KEGG [38], UCSD Recon1 [15] and Edinburgh human metabolic network [16]. The integration process was described previously [39]. The organization of metabolic networks has been described as hierarchical and modular [40]. When we perform a hierarchical clustering on the metabolic reactions in our network, its modular structure is clear (Figure 2A). This modular organization, as reported previously [41], often but not always correlates with conventional pathways (Figure 2B).

The module definition in this work is adopted from Newman and Girvan [42], [43], where a module is a subnetwork that shows more internal connections than expected randomly in the whole network. Modules are less biased than pathways, which are defined by human knowledge and conventions, and outgrown by genome-scale metabolic networks. Activity of modules may exist within and in between pathways. Deo et al [22] convincingly demonstrated the advantage of unbiased module analysis over pathways. On the other hand, pathways have built-in human knowledge, which may be more sensitive under certain scenarios. Pathway analysis and module analysis are rather complementary, and both are included in *mummichog*.

The reference metabolic network model contains both metabolites and enzymes. Since metabolomics only measures metabolites, the model is converted to a metabolite centric network for statistical analysis. Enzymes are only added later in the visualization step to aid user interpretation.

### Module analysis

Within the predefined reference metabolic network model 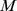, *mummichog* searches for all modules that can be built on user input data, and compute their activity scores. This process is repeated many times for the permutation data to estimate the background null distribution. Finally, the statistical significance of modules based on user data is calculated on the null distribution. The specific steps are as follows:

1. A list of “input metabolites” is tentatively computed from the significant list of *m/z* features, 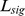. The *m/z* - metabolite matching considers all common isotopic derivatives and adducts.
2. From 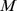, all subnetworks that can connect the input metabolites from Step 1 within one reaction are extracted. This is repeated for subnetworks within two, three and four reactions (by four steps 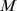 should be exhausted, see Figure 2C). These subnetworks contain both “input metabolites” and other metabolites from 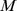.
3. Structural modules are identified within these subnetworks according to Newman's spectral split algorithm [43]. This creates many modules of different sizes.
4. Both subnetworks from Step 2 and modules from Step 3 are compiled into a set of candidate modules (graphs), 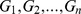. Each graph is cleaned of protruding edges if such edges do not connect input metabolites therefore not contributing to the activity score.
5. The activity score should reflect both the enrichment of “input metabolites” and the modularity. Activity score of a candidate module is computed as follows: For a subgraph 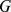, activity score
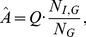
(1)where 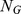 is the number of metabolites in 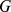, 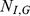 the number of “input metabolites” in 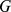, 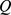 the adjusted Newman-Girvan modularity:
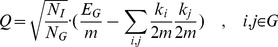
(2)where 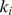 is the network degree of metabolite 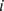, 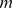 the total number of edges in the metabolic network 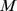, 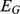 the total number of edges in 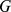, 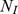 the number of “input metabolites”. The original Newman-Girvan modularity has a bias towards larger modules. The 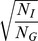 is added to reduce this bias.
6. A list of permutation features (equal length to 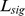) is generated by randomly sampling 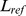. The activity scores of modules from this permutation list are computed as in Steps 1–5.
7. Repeat Step 6 many times to populate a list of scores from random modules. Using maximum likelihood estimation, these scores are modeled as a Gamma distribution (this is the null distribution), and a cumulative distribution function (CDF) is calculated.
8. The p-value for each module from input metabolites is calculated on the CDF of null distribution.

### Pathway analysis

The basic test for pathway enrichment here is Fisher's exact test (FET), which is widely used in transcriptomic analysis. The concept of FET is, when we select 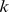 features (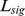) from a total of 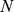 features (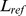), and find 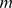 of the 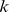 features present on a pathway of size 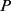, the chance of getting 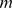 in theory can be estimated by enumerating the combinations of 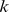, 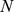 and 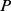.

To apply FET to an enrichment test of metabolic features on pathways, we need to understand the additional layer of complexity. Our metabolic features can be enumerated either in the *m/z* feature space or in the metabolite (true compound) space. Since metabolic pathways are defined in the metabolite space, either way needs to factor in the many-to-many mapping between *m/z* features and metabolites (Figure S1). This mapping is effectively covered in our permutation procedure, which starts from the *m/z* features and reruns the mapping every time. The overall significance of a pathway enrichment is estimated based on a method by Berriz et al [44], which ranks the p-value from real data among the p-values from permutation data to adjust for type I error. Finally, a more conservative version of FET, EASE, is adopted to increase the robustness [45]. The key idea of EASE is to take out one hit from each pathway, thus preferentially penalize pathways with fewer hits. The specific steps are as follows:

1. A list of permutation features (equal length to 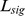) is generated by randomly sampling 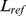. This list is mapped to a list of tentative metabolites. These metabolites are looked up in each metabolic pathway. For each pathway, a FET right-tail p-value is calculated.
2. Repeat Step 1 many times to populate a list of p-values of all pathways under all permutations (null distribution). Slightly different from Berriz et al [44], all p-values not just the minimal p-values in each permutation are used. This is because the size and organization of metabolic pathways vary greatly, a different situation from the large gene categories in Berriz et al. Using maximum likelihood estimation, these p-values are modeled as a Gamma distribution (this is the null distribution), and a cumulative distribution function (CDF) is calculated.
3. Perform Step 1 on the significant feature list 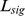, and calculate FET p-value and EASE score for each pathway. An adjusted p-value per pathway is calculated based on the EASE score and CDF from Step 2. Both FET p-value and adjusted p-value are reported for each pathway.

### Data driven activity network

**Figure 3:**
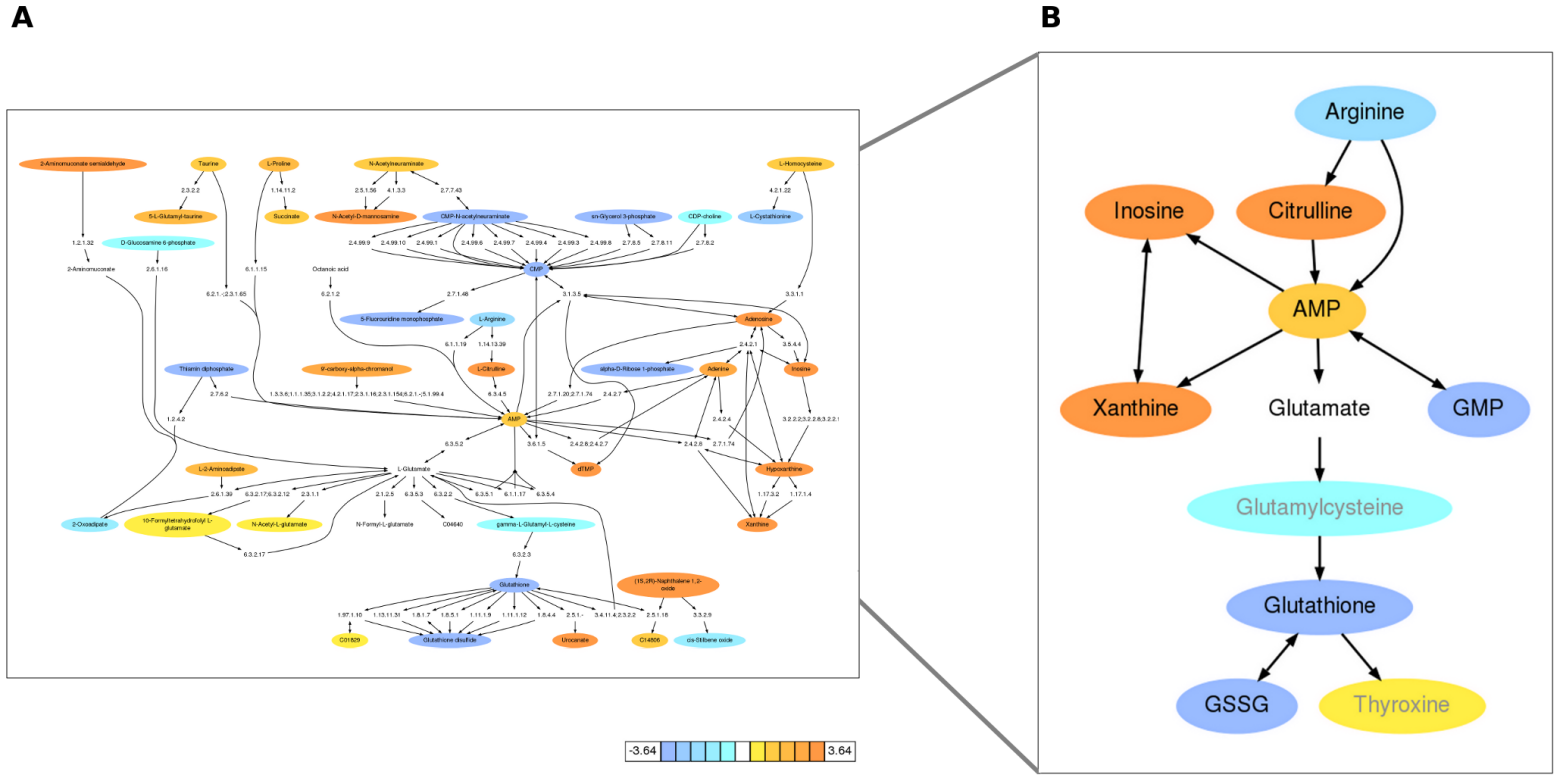
Metabolic activity network in dendritic cells stimulated by yellow fever virus. A) Prediction by *mummichog* directly from *m/z* feature tables (cell extracts after 6 hours of infection). Metabolites are colored according to log2 fold change. A high resolution copy is given in Figure S9. B) Further investigation was focused on the subnetwork on the right. Glutamate was not significantly altered, but included for network connectivity.

Both the module analysis and pathway analysis above serve as a framework to estimate the significance of functional activities. In return, the predicted metabolites in significant activities are more likely to be real. *Mummichog* collects these metabolites, and look up all their isotopic derivatives and adducts in 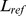. A confidence rating system is applied to filter for qualified metabolites. For instance, if both the single-charged 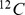 form M+H[1+] and the 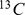 form M(C13)+H[1+] are present, this metabolite prediction carries a high confidence. All the qualified metabolites carry over their connections in the reference metabolic network, and form the “activity network” for this specific experiment (e.g. Figure 3).

The activity network gears towards a quality and clear view of user data, as modules and pathways can be redundant and fragmented. It also accentuates the activity in a specific experimental context, in contrast to the generic nature of the reference metabolic network.

We next illustrate the application of these algorithms to a novel set of immune cell activation data, and two published data sets on human urinary samples and yeast mutants.

### Application to the activation of innate immune cells

The innate immunity plays a critical role in regulating the adaptive immunity, and the field was recognized by the 2011 Nobel Prize in Physiology or Medicine [46]. According to the nature of stimuli, innate immune cells direct different downstream molecular programs, which are still under intense scientific investigation [47], [48]. In this study, we examine the metabolome of human monocyte-derived dendritic cells (moDC) under the stimulation of yellow fever virus (YF17D, a vaccine strain). We have shown previously that yellow fever virus activates multiple toll-like receptors, and induces cellular stress responses [49]–[51]. This data set is, to our knowledge, the first high throughput metabolomics on any immune cells (macrophages were previously studied by limited throughput).

The cell extracts from our activation experiment were analyzed by LC/MS metabolomics, and yielded 7,995 spectral features (denoted as 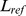) after quality control. Among them, 601 features were significantly different between the infected samples and both the baseline and time-matched mock controls (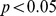, student t-test; denoted as 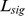). Using 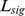 and 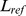, *mummichog* computes significant pathways and modules and the activity network.

**Figure 4:**
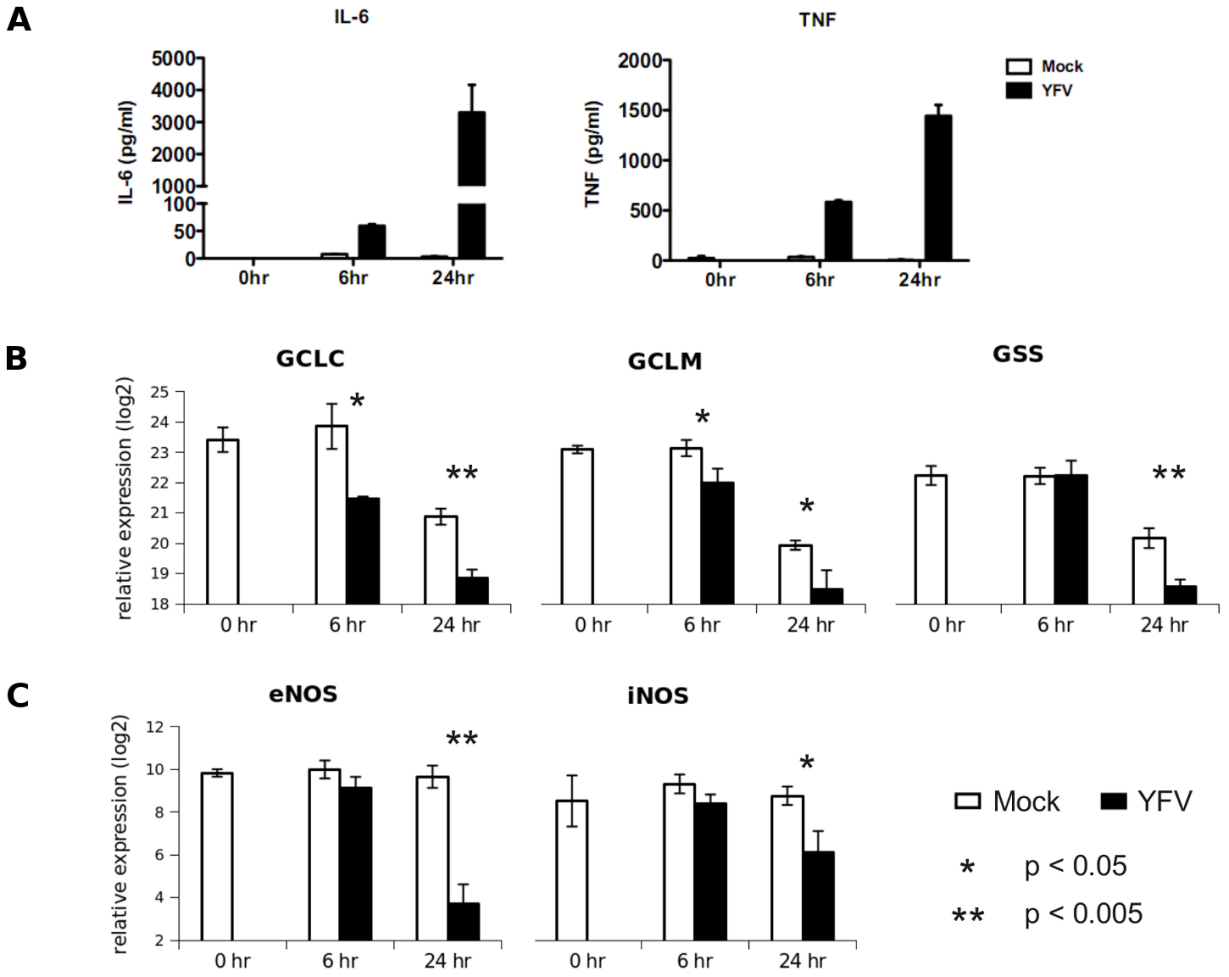
Gene expression confirms the activity network. A) Cytokines secreted after infection (ELISA) indicate the activation of innate immune programs. B) Down-regulation of transcripts of GCLC, GCLM (subunits of gamma-glutamylcysteine synthetase) and GSS (glutathione synthetase), the key enzymes for glutathione synthesis. C) Nitric oxide has feedback inhibition on the expression of eNOS and iNOS (nNOS was not detected). Gene expression was assayed by quantitative RT-PCR. Infected samples were compared to mocks by student's t-test (n = 3).

**Figure 5:**
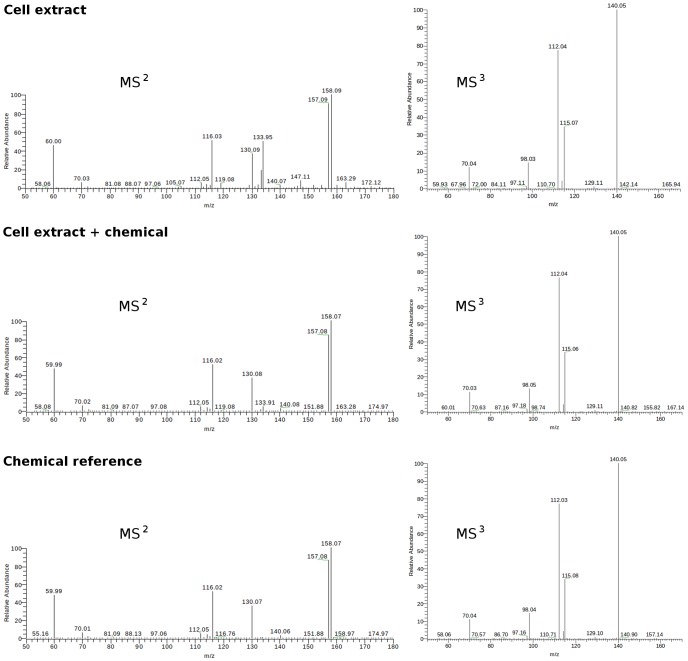
Identification of metabolites by tandem mass spectrometry. Arginine is shown as an example while the full data are given in Figure S3. From top to bottom: the fragmentation pattern (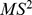 followed by 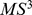 on peak 157) from biological sample, from biological sample spiked with authentic chemical and from authentic chemical reference.

Viral infection induced a massive shift of metabolic programs in moDCs (pathways in Table S1, modules in Figure S2). The predicted activity network is shown in Figure 3A, and we will focus our investigation on a small subnetwork (Figure 3B), which includes the metabolisms of nucleotides, glutathione/glutathione disulfide and arginine/citrulline. Nucleotides are required for viral replication, and the hijacking of host nucleotide metabolism by virus has been well described [52]–[54]. Glutathione is best known as intracellular antioxidant, where it is oxidized to glutathione disulfide (GSSG). However, our data show that both glutathione and GSSG are depleted in activated moDCs, departing from this conventional wisdom. The across-the-board depletion is consistent with the down-regulation of genes for glutathione synthesis (Figure 4B). Our data support the notion that glutathione is released by dendritic cells and conditions the extracellular microenvironment during their interaction with T cells [55]–[57]. Arginine is known to be an important regulator in innate immune response [58], [59]. Arginine metabolism can lead to two pathways: to ornithine (catalyzed by arginase) or to citrulline (catalyzed by nitric oxide synthase). The decrease of arginine and increase of citrulline suggests the latter pathway, which is the main reaction of producing intracellular nitric oxide. We indeed detected the inhibition of eNOS and iNOS expression later (Figure 4C), a well documented feedback effect by nitric oxide [60]. We also performed tandem mass spectrometry on the metabolites in Figure 3B, using authentic chemicals as references. All the metabolites, except glutamylcysteine and thyroxine, were confirmed (Figure 5, Figure S3).

The depletion of arginine and accumulation of citrulline in moDC was also triggered by dengue virus but not by lipopolysaccharide (LPS, Figure S4). It is known that iNOS is induced in dendritic cells by LPS but not by virus [47], [61]. Our data suggest a different nitric oxide pathway in viral infection, driven by constitutive nitric oxide synthases. The intracellular nitric oxide has a fast turnover and we did not detect its accumulation by fluoremetric assays (data not shown). We previously demonstrated that the phosphorylation of EIF2A was induced by YF17D [50]. An upstream mechanism is now suggested by this metabolomic experiment, as both the production of nitric oxide and depletion of arginine induce the activity of EIF2A kinases [62].

### Evaluation on human urine data and yeast data

The nature of metabolomics data often varies by platforms and sample types. We thus extend our *mummichog* approach to two published data sets on human urinary samples [63] and on yeast cell extracts [64]. Both data sets carry metabolite annotation by the original authors, which can be used to evaluate the prediction by *mummichog*.

**Figure 6:**
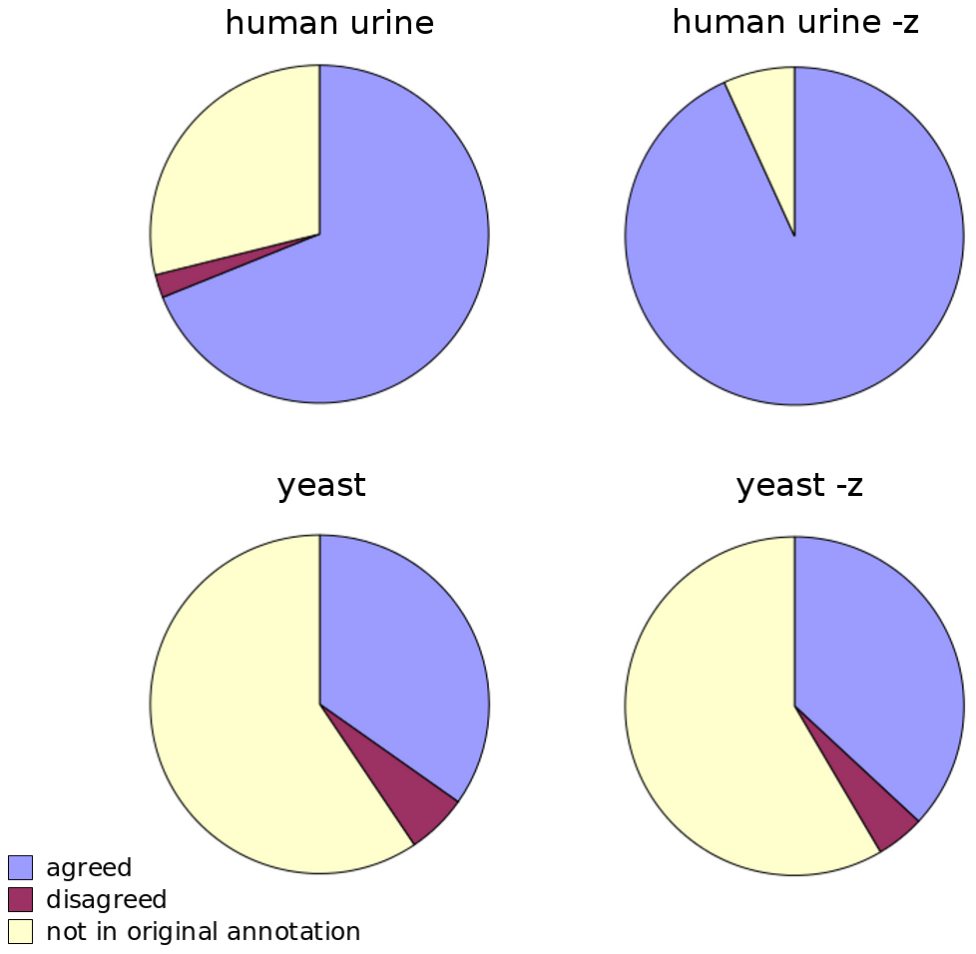
Application of mummichog to additional data sets. Metabolite prediction by *mummichog* is in good agreement with annotation in the original studies, 97% for the human urine data [63] and 86% for the yeast data [64]. The metabolites not in the original annotation (yellow) can not be compared. The “-z” option in *mummichog* enforces the presence of primary ion (M+H[+] for positive mode, M−H[−] for negative mode). This shifts the coverage in the huamn data set, but not much for the yeast data of limited annotation.

The human urinary data contained both formal identification by matching to local library of chemical references and putative identification by combining multiple public resources [63]. We used *mummichog* to investigate the gender difference in this data set, and predicted an activity network of 45 metabolites. Among them, 13 were not found in the original annotation. For the remaining metabolites, 97% (31/32) were agreed between *mummichog* and the original annotation (Figure 6). There is an option in *mummichog* to enforce the presence of M+H[+] form (for positive mode, M−H[−] for negative mode) in metabolite prediction. With this option, 3 out of 44 metabolites were not in the original annotation, and the remaining 41 metabolites were in 100% agreement.

The *mummichog* algorithms are not tied to a specific metabolic model. We adopted the yeast metabolic model from BioCyc database [11] for the yeast data [64], to predict an activity network contrasting mutant and wild type strains. This data set was only annotated for 101 metabolites through the authors' local library. As a result, the majority of metabolites in the predicted network by *mummichog* were not found in the original annotation. Out of the remaining 28 metabolites, 24 (86%) were agreed between mummichog and the original annotation (Figure 6). Enforcing the presence of primary ion M−H[−] (data collected in negative ion mode) had little effect to the result, since the original annotation was already biased to metabolites that are ionized easily.

These results show that the prediction by *mummichog* is robust cross platforms and sample types.

## Discussion

We have described the computational method of *mummichog* and its application to three case studies. While the description was intended to be concise, computational metabolomics is complicated and facing challenges from many fronts. Quantification and feature selection, the steps upstream of *mummichog*, can still be problematic. Data from *in vivo* studies often involve multivariate analysis, where validation is particularly challenging. In this section, we discuss several confounding factors that are relevant to our approach.

### Impact of metabolic models

Critical to the success of *mummichog* is the integration of genome-scale metabolic models. We have used in this study a recent human metabolic model. An alternative human model from BioCyc [11] produced comparable results (Figure S6). The coverage of the models in all three case studies is shown in Table 1. These genome-scale metabolic models are more extensive than conventional pathways, and were shown to capture activities in between pathways [22]. The pathway organizations differ between the two human models, as the BioCyc model tends to use smaller pathways. This creates some model dependency in the pathway analysis, but little effect to the “activity network”, as *mummichog* is more network centric. The two test cases in Figure 6 also indicate that these models tend to capture more information than conventional annotations.

**Table 1:** How metabolomics data match metabolic models.

	human model	human model (BioCyc)	yeast model (BioCyc)
number of reactions	4204	2091	1034
metabolite edges	18504	14100	6962
number of metabolites	3565	1536	811
number of metabolites with exact mass	2016	1308	742
case moDC: reference features	7713	7713	
case moDC: tentatively matched metabolites	1384	874	
case urine: reference features	10248	10248	
case urine: tentatively matched metabolites	1453	930	
case yeast: reference features			5366
case yeast: tentatively matched metabolites			533

The top section gives statistics of each of the three metabolic models. The bottom section gives the specific number of measured features and tentative metabolite matches in each study case. These tentative matches contain excessive ambiguity, which mummichog aims to resolve.

However, as mentioned earlier, the new data from metabolomic studies are yet to be integrated into these genome-scale metabolic models. For example, a number of metabolites in metabolomics databases [36], [65], [66] are not in any of these metabolic models. In general, the features from a high resolution profiling experiment by far exceed the current annotations in metabolite databases. This leads to a *de facto* filtering when data are run on *mummichog* (similiar situation in database searches). Meanwhile, the features that can be mapped to the current metabolic model are more likely to be biologically relevant. This “filtering” is pertinent to the metabolic model, not to *mummichog* algorithms - *mummichog* still has to choose the true metabolites from multiple possible candidates (Figure S1B).

It will be an important future direction to advance metabolic modeling with the chemical data. We also expect the metabolic models to improve on lipid annotation, physiological context and tissue specificity.

### Robust design of the algorithms

As lessons learned from transcriptomics, pathway and network analysis not only provides functional context, but also the robustness to counteract noises at individual feature level, which are commonly seen in omics experiments. Similarly, the prediction on activity by *mummichog* is tolerant to errors at individual feature level. In the moDC data, we chose 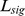 by a cutoff value 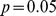. When we vary this cutoff from 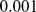 to 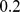, the program returned networks of a stable set of metabolites (Figure S7).

The module finding procedure in the program was designed to extensively sample subnetwork structures. Among the modules will be many variations, but the subsequenct “activity network” will collapse on stable results. In deed, we tested an alternative algorithm of modularization [67], and it returned almost identical predicted networks, in spite of moderately different intermediate modules (Figure S8).

In theory, there are merits to incorporate a statistical matrix from the feature selection step prior to *mummichog*'s analysis and mass flow balance of metabolic reactions [22], [68]. While these are appealing directions for future research, the current version of *mummichog* confers some practical robustness, such as tolerance to technological noise and biological sampling limitation. For example, mass balance is almost impossible within serum or urine samples, because the reactions producing these metabolites are likely to occur in other tissues.

The number of overlap metabolites is used in the enrichment calculation in both module analysis and pathway analysis. Sometimes, a single *m/z* feature may match to several metabolites in the same module/pathway, inflating the overlap number. Thus, *mummichog* always compares the number of overlap metabolites and the number of corresponding *m/z* features, and uses the smaller number for enrichment calculation, since the smaller number is more likely to be true.

The size of each metabolic pathway is defined by the number of metabolites in the pathway. *mummichog* uses only the metabolites that can be matched in 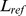 to define a pathway size, because this reflects the analytical coverage of the experiment and 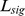 is confined by the same coverage. Overall, *mummichog* uses the whole feature list from an experiment for resampling, therefore the computation of statistical significances effectively circumvents analytical biases.

### Acceleration of untargeted metabolomics

In spite of the fantastic progress in mass spectrometry, these are the early days of metabolomics. Effective computational integration of resources, the combination of cheminformatics and bioinformatics, will greatly benefit the field [69], [70]. As data accumulate, further method refinement will become possible.

*Mummichog* presents a practical solution of one-step functional analysis, bypassing the bottleneck of upfront metabolite identification. It trades off some sensitivity in the conventional approach for the much accelerated work flow of high throughput LC/MS metabolomics. *Mummichog* is not designed to replace tandem mass spectrometry in metabolite identification. It is the biological activity not metabolites *per se* that *mummichog* predicts. Even with some errors on individual metabolites, as long as the biology is pinpointed to a subnetwork structure, investigators can focus on a handful of validations, steering away from the lengthy conventional work flow.

In conclusion, we have demonstrated that *mummichog* can successfully predict functional activity directly from a spectral feature table. This benefits from the convergence of genome-scale metabolic models and metabolomics. *Mummichog* will continue to improve as the metabolic network models evolve. We expect this method to greatly accelerate the application of high throughput metabolomics. The *mummichog* software is available at http://atcg.googlecode.com.

## Methods

### Cell culture and infection

Human peripheral blood mononuclear cells (PBMCs) were isolated from Buffy coats by separation over a Lymphoprep gradient. CD14+ monocytes were isolated from the PBMCs with MACS beads (Miltenyi Biotec, Auburn, CA) and cultured for 7 days with 20 ng/ml GM-CSF and 40 ng/ml IL-4 (Peprotech, Rocky Hill, NJ). MoDCs were then harvested, washed twice and resuspended in serum-free medium.

MoDCs (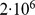) were stimulated in triplicate in 48-well plates in a 200 µL volume with Yellow Fever virus (M.O.I. of 1), or mock infected. After 2 hrs, 800 µL of 10% FBS-RPMI was added to all wells. MoDCs were harvested at 6 hr or 24 hr after infection and centrifuged. Supernatants were aspirated, and dry cell pellets were frozen at −80°*C*. Supernatants of moDC cultures were assayed for the concentration of IL-6 and TNF using ELISA kits (BD, San Diego, CA). Three biological replicates were used for LC/MS and QPCR.

### Mass spectrometry

Full scan LC/MS (*m/z* range 85–2000) was performed essentially as previously described [8]. Cell extracts or supernatants were treated with acetonitrile (2∶1, v/v) and centrifuged at 14,000× g for 5 min at 4°*C* to remove proteins. Samples were maintained at 4°*C* in an autosampler until injection. A Thermo Orbitrap-Velos mass spectrometer (Thermo Fisher, San Diego, CA) coupled with anion exchange chromatography was used for data collection, via positive-ion electrospray ionization (ESI). Metabolites of interest were identified by tandem mass spectrometry on a LTQ-FTMS, where the biological sample, biological sample spiked in with authentic chemical and authentic chemical reference were run sequentially. The 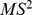 and 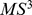 were done in the ion trap of the LTQ-FTMS, with an isolation width of 1 amu and a normalized collision energy of 35 eV.

The LC/MS data were processed with apLCMS program [25] for feature extraction and quantification. Significant features were also verified by inspecting the raw data (Figure S5). Features were removed if their intensity is below 10,000 in every sample class. Missing intensity values were imputed to 1000. The intensities were log2 transformed. Low quality features were further filtered out if their averaged in-class coefficient of variation was greater than 0.2. Averaged ion intensity over three machine replicates was used for subsequent analysis. These 7,995 features constituted the reference list 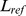. No normalization was used because total ion counts in all samples were very similar. Student's t-test was used to compare infected samples (at 6 hr) versus mock infections (at 6 hr), and infected samples (at 6 hr) versus baseline controls (at 0 hr). Features with 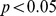 in both tests were included in the significant list 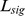. The feature table, 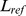, 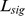 and predictions are given in Dataset S1.

### Gene expression by QPCR

For gene expression quantification, mRNA was extracted by RNeasy Mini Kit (Qiagen) according to manufacturer's protocol, where the cell lysate was homogenized by QIAshredder spin columns. Reverse transcription was performed with SuperScript III reverse transcriptase and oligo-dT (Invitrogen) according to manufacturer's recommendation. Real-time PCR was performed on a MyiQ Icycler (BioRad), using SYBR Green SuperMix (Quanta Biosciences). The PCR protocol used 95°*C* 3 mins; 40 cycles of 95°*C* 30 seconds and 60°*C* for 60 seconds. The amplicons were verified by melting curves. Quantafication was performed by the 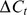 method, normalized by 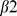 microglobulin levels. The primer sequences are given in Table S2.

### Analysis of human urine and yeast data

Data on human urinary samples [63] were retrieved from MetaboLights server [71]. The positive ion feature table for study “439020” contained 14,720 features. A feature is only included if its ion intensity is above 100,000 in 5 or more samples. This leaves 11,086 features, which consist 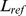 for this study. Data were normalized by total ion counts. We next compared the metabolite difference between females (8 samples of low testosterone glucuronide level) and males (11 samples of high testosterone glucuronide level). 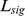 is consisted of 524 features (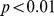 by student t-test). The original authors annotated 3,689 metabolite features, and their annotation was used to compare with the prediction by *mummichog*.

The yeast data [64] were downloaded from MAVEN website [32] in mzXML format. Feature extraction was performed in MAVEN through two approaches: targeted approach and untargeted approach. The targeted approach used chemical library from the same lab and produced 177 annotated features, which corresponded to 101 metabolites. The untargeted approach extracted 6318 features without annotation. After the same processing procedure as in our moDC data, 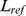 contained 5707 features. We thus used *mummichog* to predict on the untargeted data, and compared the result to MAVEN annotation. The 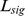 consisted of 426 features that were significantly different between the prototrophic wild type and the auxotrophic mutant (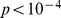 by student t-test). The yeast metabolic model was compiled from BioCyc data [11].

## Supporting Information

Dataset S1
**Metabolomics feature table and** ***mummichog*** **predictions.** This file contains LC/MS Metabolomics feature table from the moDC experiment, 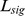, 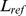 and predicted metabolites in the activity networks of moDC activation. Metabolite predictions on the human urine data and yeast data are also included.(ZIP)

Figure S1 **Many-to-many relationship in m/z feature to metabolite matching.** A) Among m/z features in Lref, about 1400 can be matched to various metabolites. B) For the 77 metabolites in the activity network (Figure 3A), on average, about five metabolites share the same m/z features. *Mummichog* chooses the most likely metabolites based on their network activities and spectral patterns.
(TIF)

Figure S2
**Example of module analysis.** Five significant modules were identified (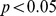, default parameters) in moDC infected by yellow fever virus for 6 hours. This shows module 1, with 34 metabolites, p = 5.19E-7. This is one of the figures automatically generated by mummichog, metabolites colored by log2 fold change, with connecting enzymes shown as EC numbers.(TIF)

Figure S3
**Tandem mass spectrometry data for the metabolites in** **Figure 3B** **.** 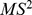 on the left and 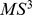 on the right. Each run contained sequentially: top) the candidate metabolite in cell extract, middle) cell extract spiked in with pure chemical reference, bottom) pure chemical reference alone. 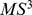 data were also obtained for major 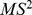 peaks except for Inosine. Elution peaks of these samples in liquid chromatography were also matched.(PDF)

Figure S4 **The depletion of arginine and accumulation of citrulline was also observed in moDCs stimulated by dengue virus, but not by LPS.** Cells and supernatant were collect after 6 hours of infection and measured by LC/MS. Representative of two experiments.
(TIF)

Figure S5 **Verification of metabolite quantification.** Glutathione is used as an example (m/z 308.0896, M+H[1+]). Left panel shows intensities of three biological replicates. The arrows point to the corresponding signals in plots of LC retention time (x-axis) vs m/z (y-axis), where a large peak is seen in YF17D sample but missing in Mock sample. In the LC-m/z plots, each dot represents a raw data point in a single scan; warmer color codes for higher ion intensity.
(TIF)

Figure S6 **Validation of** ***mummichog*** **on the human urine data set (Roux et al 2012), using metabolic model from BioCyc database.** Mummichog predicts a network of 60 metabolites, in which 19 are not found in the original annotation. Thirty nine of the remaining 41 are agreed to the original annotation. By enforcing the presence of M+H[+] ion (-z option), the predicted network contains 43 metabolite. The numbers of agreed to and not in the original annotation are 39 and 4, respectively.
(TIF)

Figure S7
**The prediction of** ***mummichog*** **is robust from the cutoff of input significant features,** 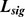 **.** The x-axis shows the different number of input features by varying p-values. The 601 features by 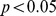 were presented in the main text, and are used as reference here. The y-axis shows the number of metabolites in predicted networks. All blue bars show the number of overlap metabolites in predicted networks, compared to those from 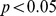.(TIF)

Figure S8 **Details of module analysis.** Using the moDC data with default parameters, *mummichog* produces 22 modules from user data and 2406 random modules by permutation. A) shows the distribution of all module sizes. B) Black horizontal bars show the distribution of activity scores from random modules, and the black line is the fitted Gamma distribution. The red vertical bars show the activity scores of modules from user data, where those on the right are more statistically significant. C) and D) follow the same format as A) and B), but use an alternative algorithm for module finding [67], which produces 84 modules from user data and 5097 random modules by permutation.
(TIF)

Figure S9 **High resolution copy of** **Figure 3A** **.**
(TIF)

Table S1 **Pathway analysis by mummichog revealed a number of pathways activated in the infection of moDCs by yellow fever virus.** Activities in many of these pathways are also identified in the significant network modules.
(PDF)

Table S2 **Primer sequences and references for quantitative PCR.**
(PDF)
