# Supplementary figures and images for: Predicting Network Activity from High Throughput Metabolomics
Source: PLoS Comput Biol. 2013 Jul 4;9(7):e1003123. doi: 10.1371/journal.pcbi.1003123 (PMC3701697; doi:10.1371/journal.pcbi.1003123)

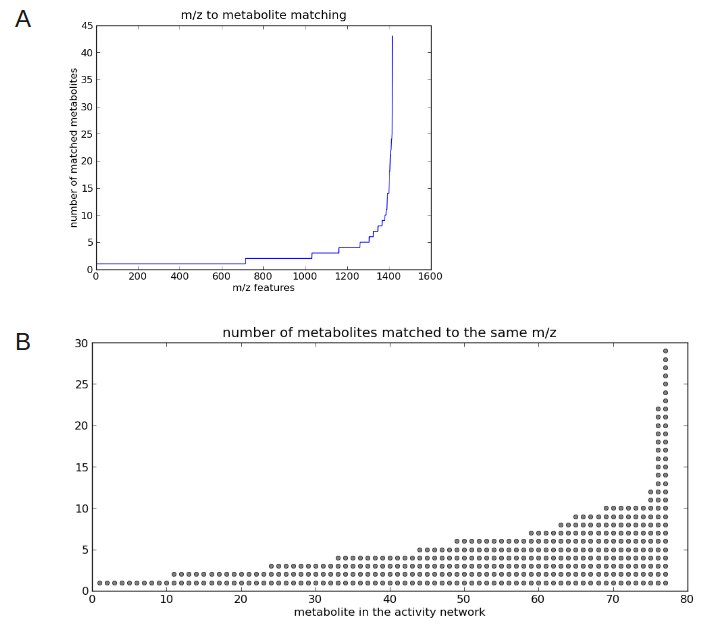

Supplement: Figure S1 — Many-to-many relationship in m/z feature to metabolite matching. A) Among m/z features in Lref, about 1400 can be matched to various metabolites. B) For the 77 metabolites in the activity network (Figure 3A), on average, about five metabolites share the same m/z features. Mummichog chooses the most likely metabolites based on their network activities and spectral patterns. (TIF) [file pcbi.1003123.s002.tif]

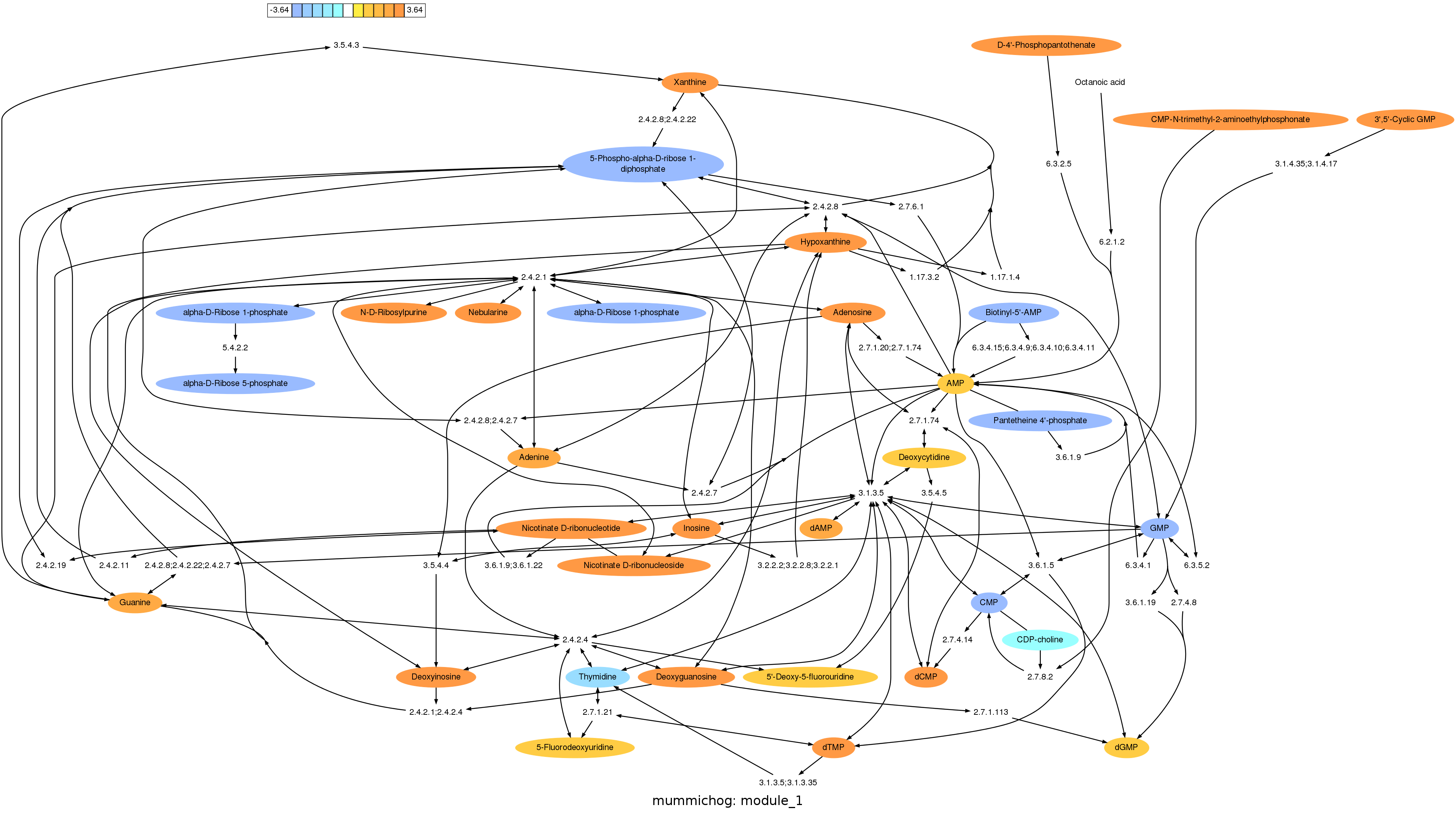

Supplement: Figure S2 — Example of module analysis. Five significant modules were identified (, default parameters) in moDC infected by yellow fever virus for 6 hours. This shows module 1, with 34 metabolites, p = 5.19E-7. This is one of the figures automatically generated by mummichog, metabolites colored by log2 fold change, with connecting enzymes shown as EC numbers. (TIF) [file pcbi.1003123.s003.tif]

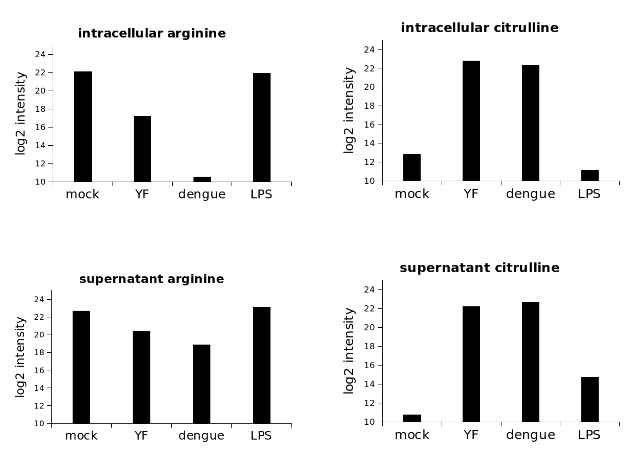

Supplement: Figure S4 — The depletion of arginine and accumulation of citrulline was also observed in moDCs stimulated by dengue virus, but not by LPS. Cells and supernatant were collect after 6 hours of infection and measured by LC/MS. Representative of two experiments. (TIF) [file pcbi.1003123.s005.tif]

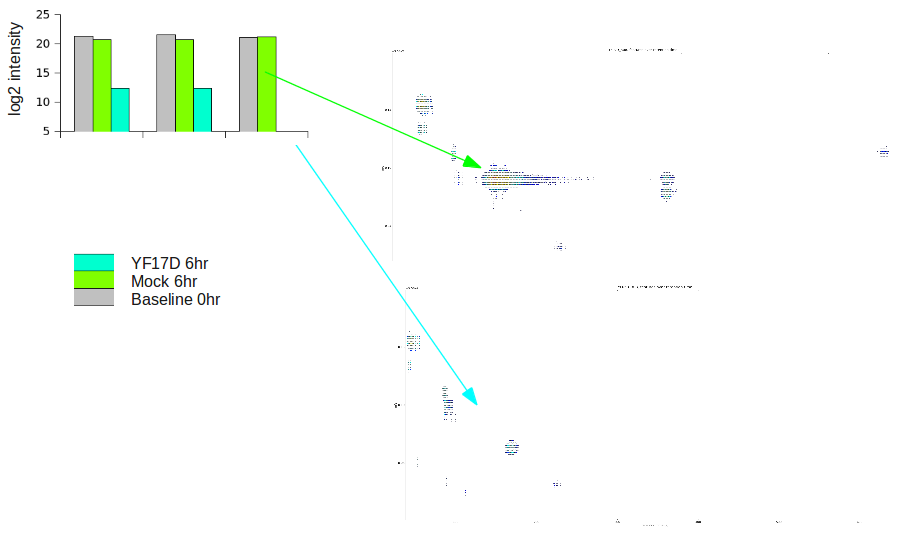

Supplement: Figure S5 — Verification of metabolite quantification. Glutathione is used as an example (m/z 308.0896, M+H[1+]). Left panel shows intensities of three biological replicates. The arrows point to the corresponding signals in plots of LC retention time (x-axis) vs m/z (y-axis), where a large peak is seen in YF17D sample but missing in Mock sample. In the LC-m/z plots, each dot represents a raw data point in a single scan; warmer color codes for higher ion intensity. (TIF) [file pcbi.1003123.s006.tif]

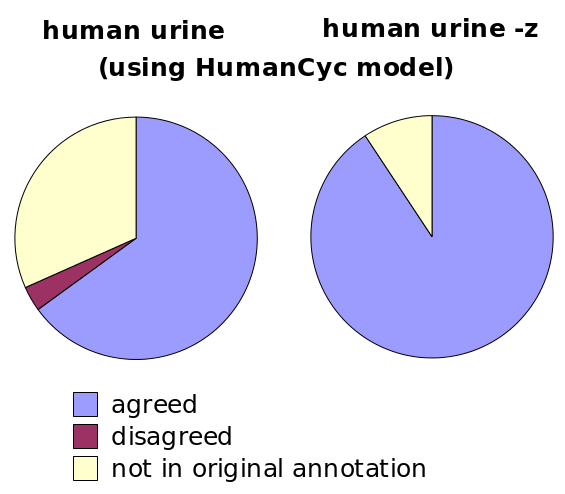

Supplement: Figure S6 — Validation of mummichog on the human urine data set (Roux et al 2012), using metabolic model from BioCyc database. Mummichog predicts a network of 60 metabolites, in which 19 are not found in the original annotation. Thirty nine of the remaining 41 are agreed to the original annotation. By enforcing the presence of M+H[+] ion (-z option), the predicted network contains 43 metabolite. The numbers of agreed to and not in the original annotation are 39 and 4, respectively. (TIF) [file pcbi.1003123.s007.tif]

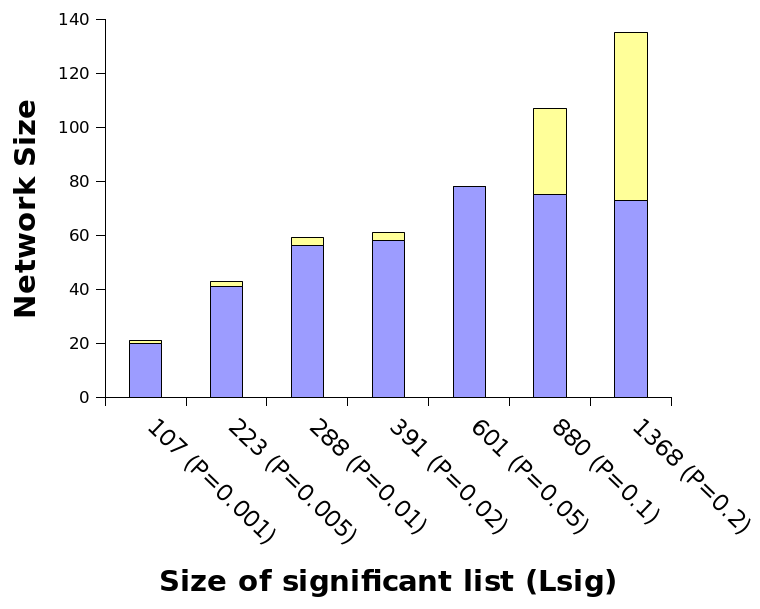

Supplement: Figure S7 — The prediction of mummichog is robust from the cutoff of input significant features, . The x-axis shows the different number of input features by varying p-values. The 601 features by were presented in the main text, and are used as reference here. The y-axis shows the number of metabolites in predicted networks. All blue bars show the number of overlap metabolites in predicted networks, compared to those from . (TIF) [file pcbi.1003123.s008.tif]

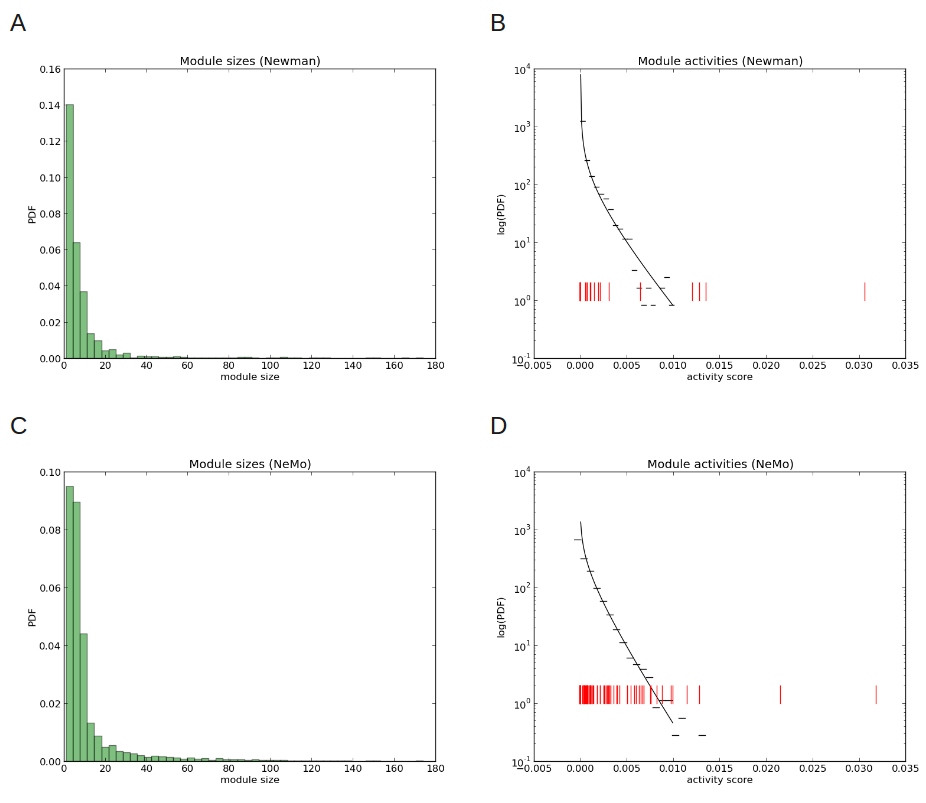

Supplement: Figure S8 — Details of module analysis. Using the moDC data with default parameters, mummichog produces 22 modules from user data and 2406 random modules by permutation. A) shows the distribution of all module sizes. B) Black horizontal bars show the distribution of activity scores from random modules, and the black line is the fitted Gamma distribution. The red vertical bars show the activity scores of modules from user data, where those on the right are more statistically significant. C) and D) follow the same format as A) and B), but use an alternative algorithm for module finding [67], which produces 84 modules from user data and 5097 random modules by permutation. (TIF) [file pcbi.1003123.s009.tif]

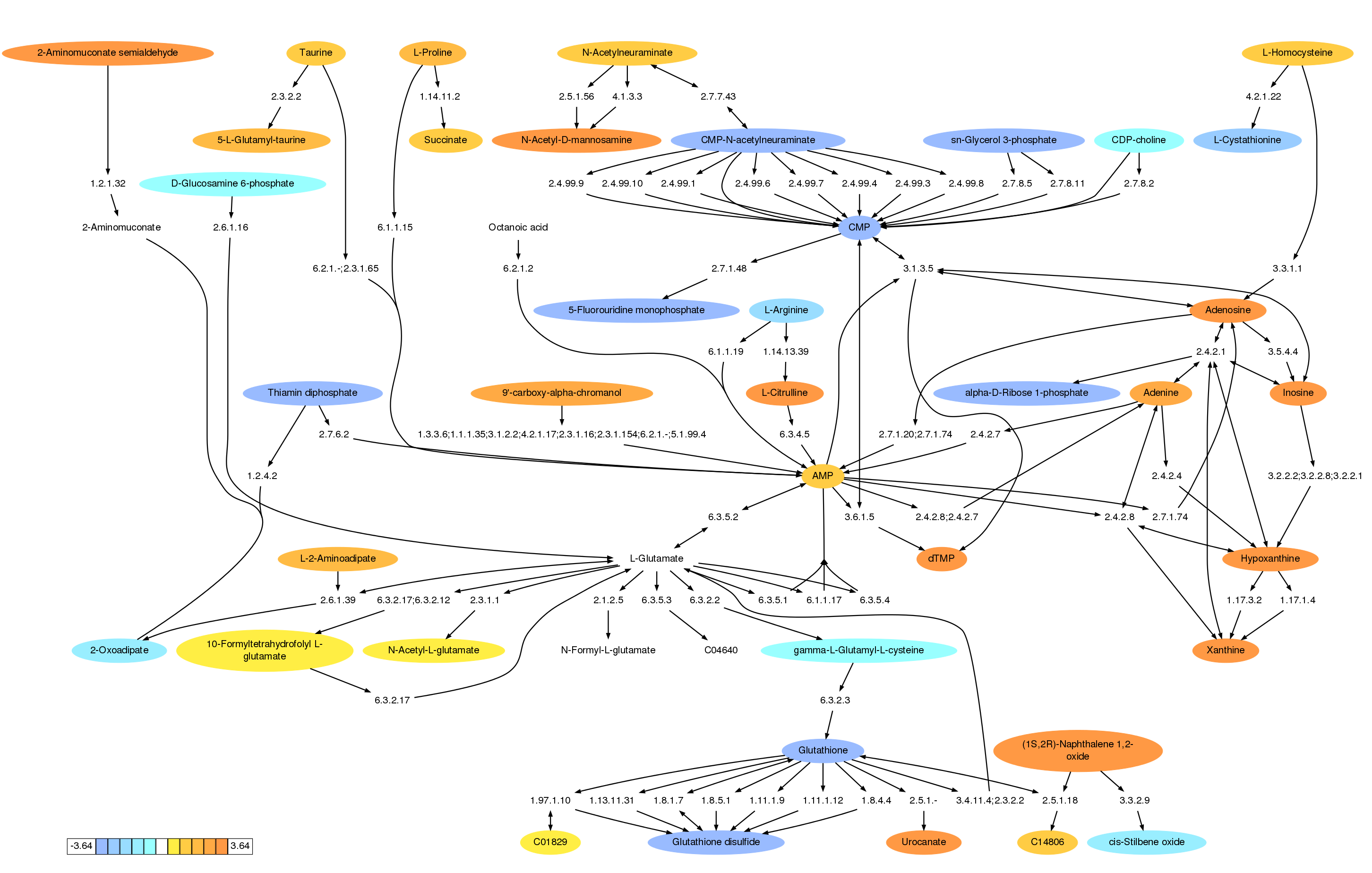

Supplement: Figure S9 — High resolution copy of Figure 3A . (TIF) [file pcbi.1003123.s010.tif]
