# Supplementary material for: Predicting Network Activity from High Throughput Metabolomics
Source: PLoS Comput Biol. 2013 Jul 4;9(7):e1003123. doi: 10.1371/journal.pcbi.1003123 (PMC3701697; doi:10.1371/journal.pcbi.1003123)
Supplement: Figure S3 — Tandem mass spectrometry data for the metabolites in Figure 3B . on the left and on the right. Each run contained sequentially: top) the candidate metabolite in cell extract, middle) cell extract spiked in with pure chemical reference, bottom) pure chemical reference alone. data were also obtained for major peaks except for Inosine. Elution peaks of these samples in liquid chromatography were also matched. (PDF) [file pcbi.1003123.s004.pdf]

Arginine M+H[1+], m/z 175.1184

cell extract

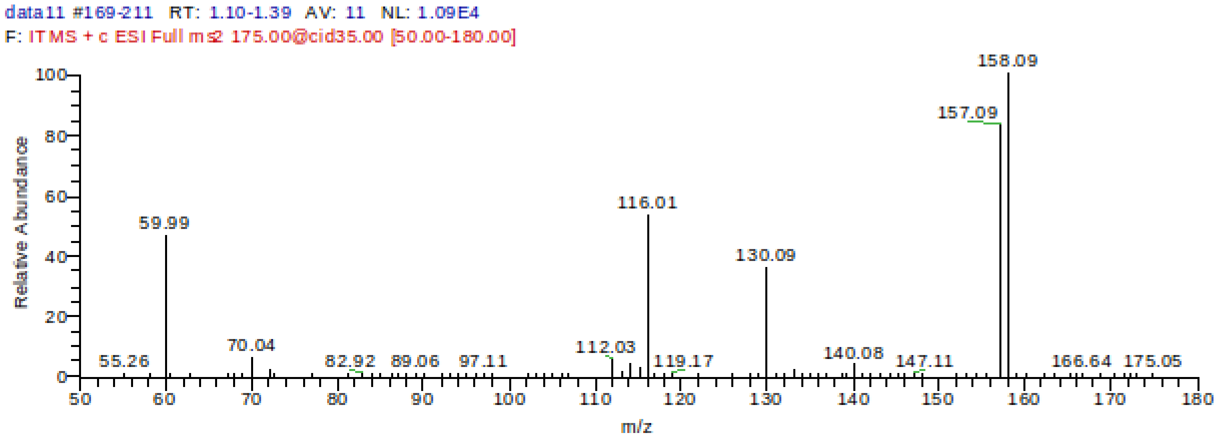

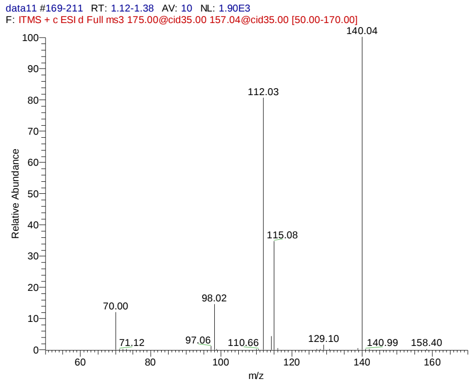

cell extract
+
chemical

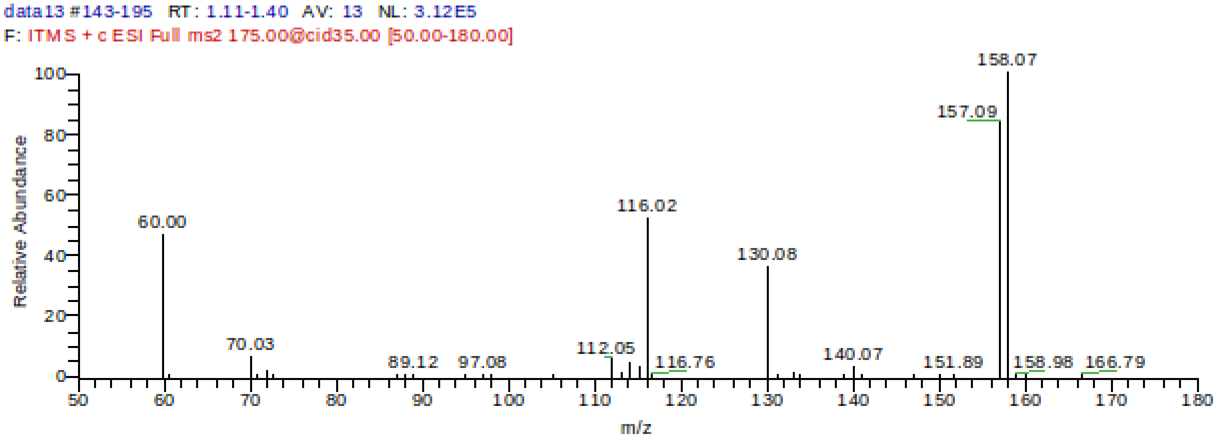

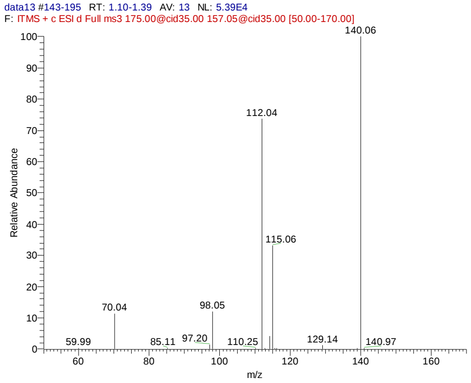

chemical
reference

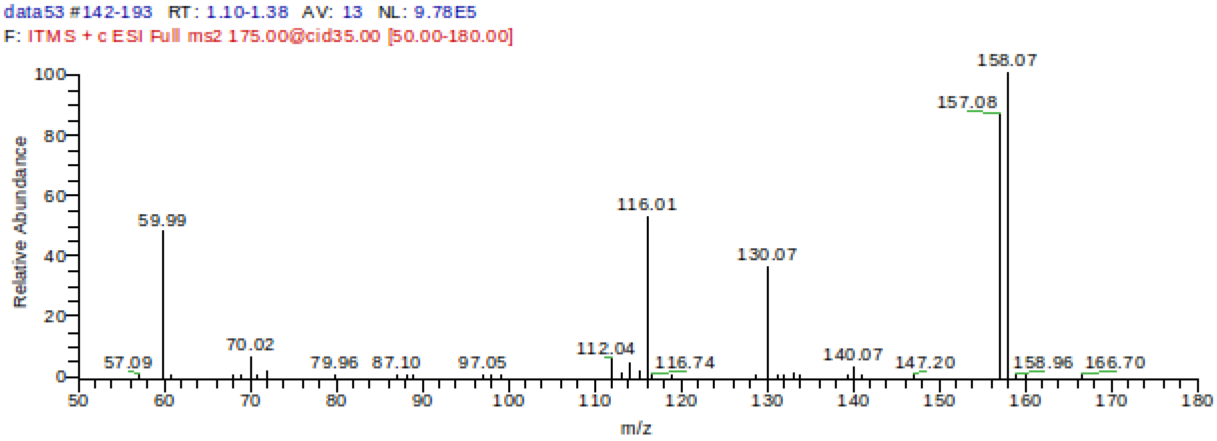

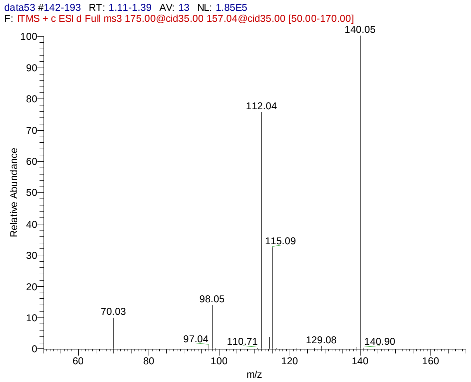

Citrulline M+H[1+], m/z 176.1022

cell extract

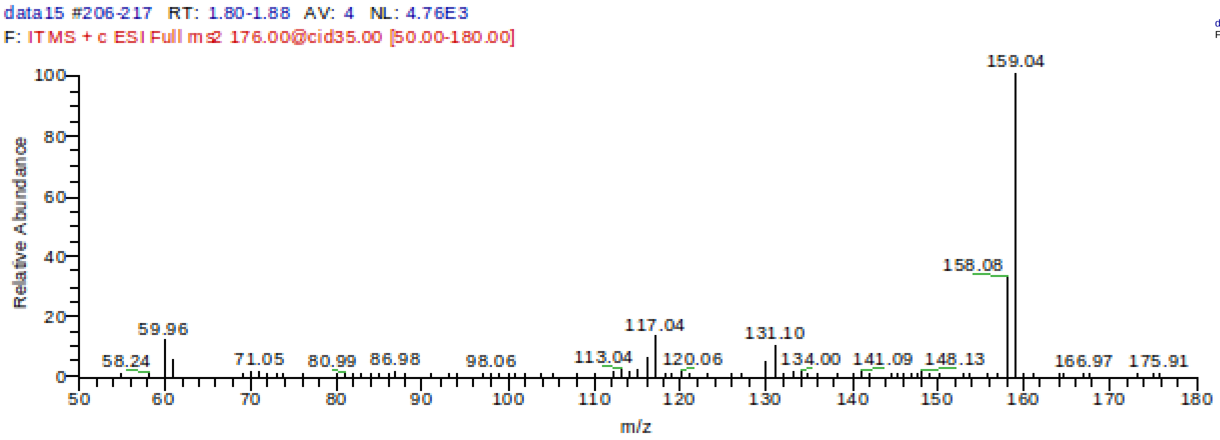

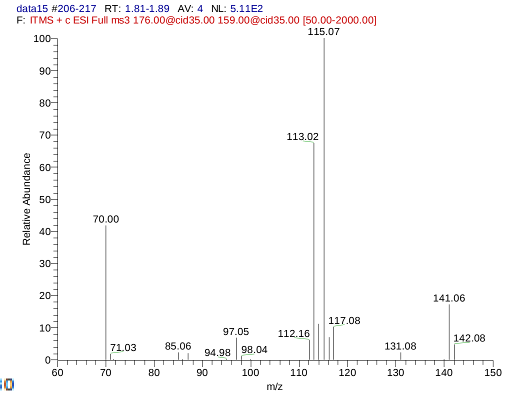

cell extract
 +
chemical

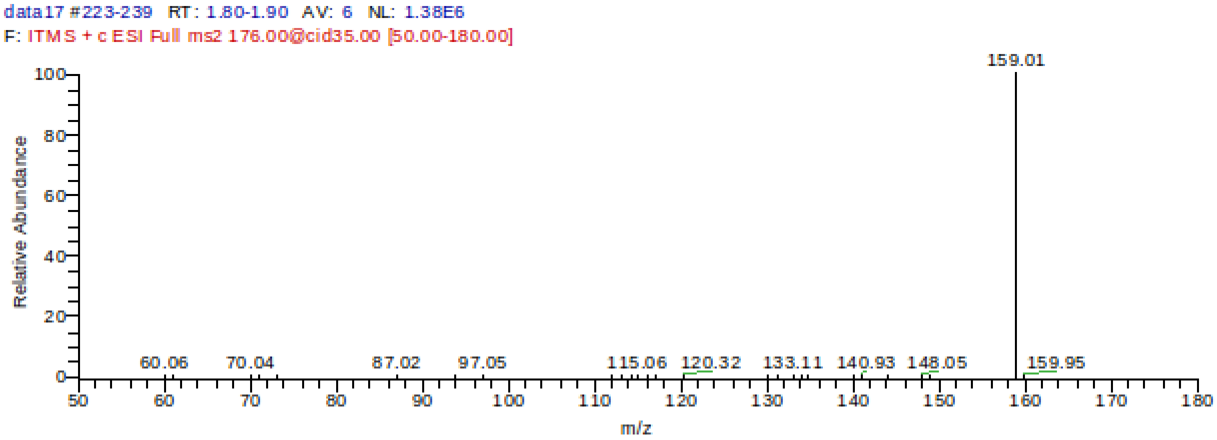

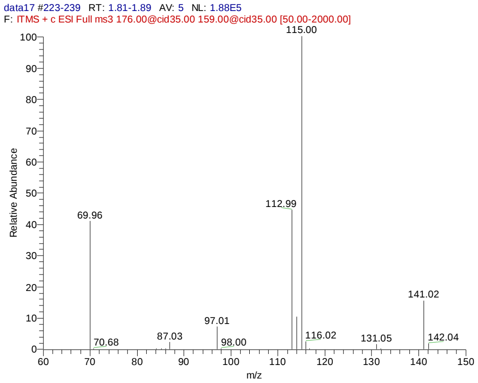

chemical
reference

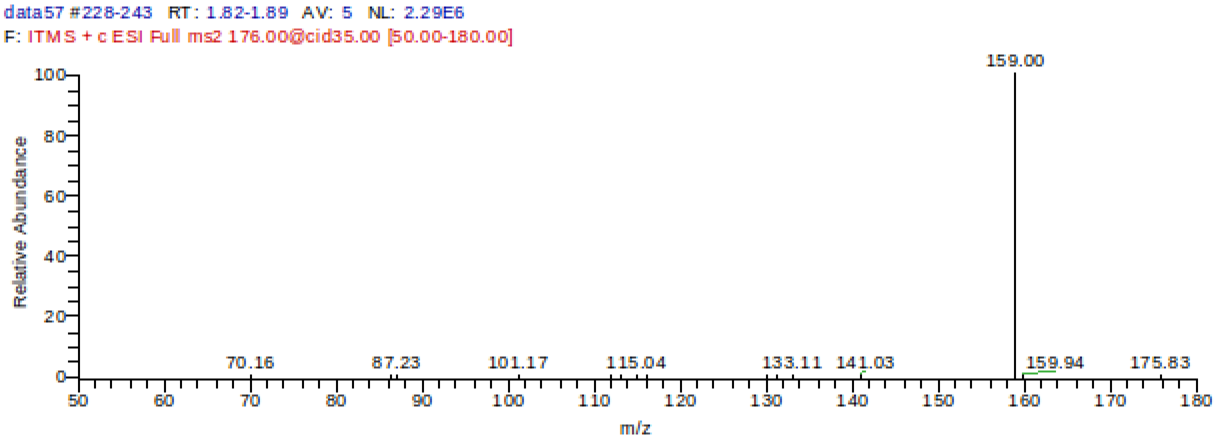

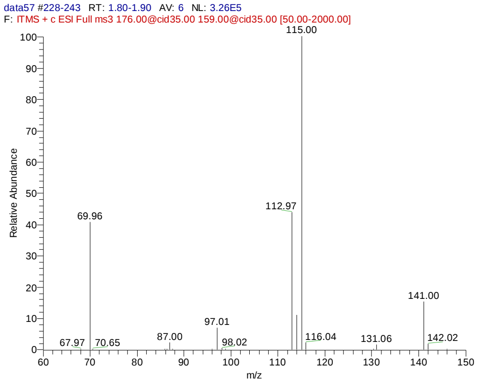

Supplementary Figure 3, ctd.

AMP M+H[1+], m/z 348.0698

cell extract

cell extract
+
chemical

chemical
reference

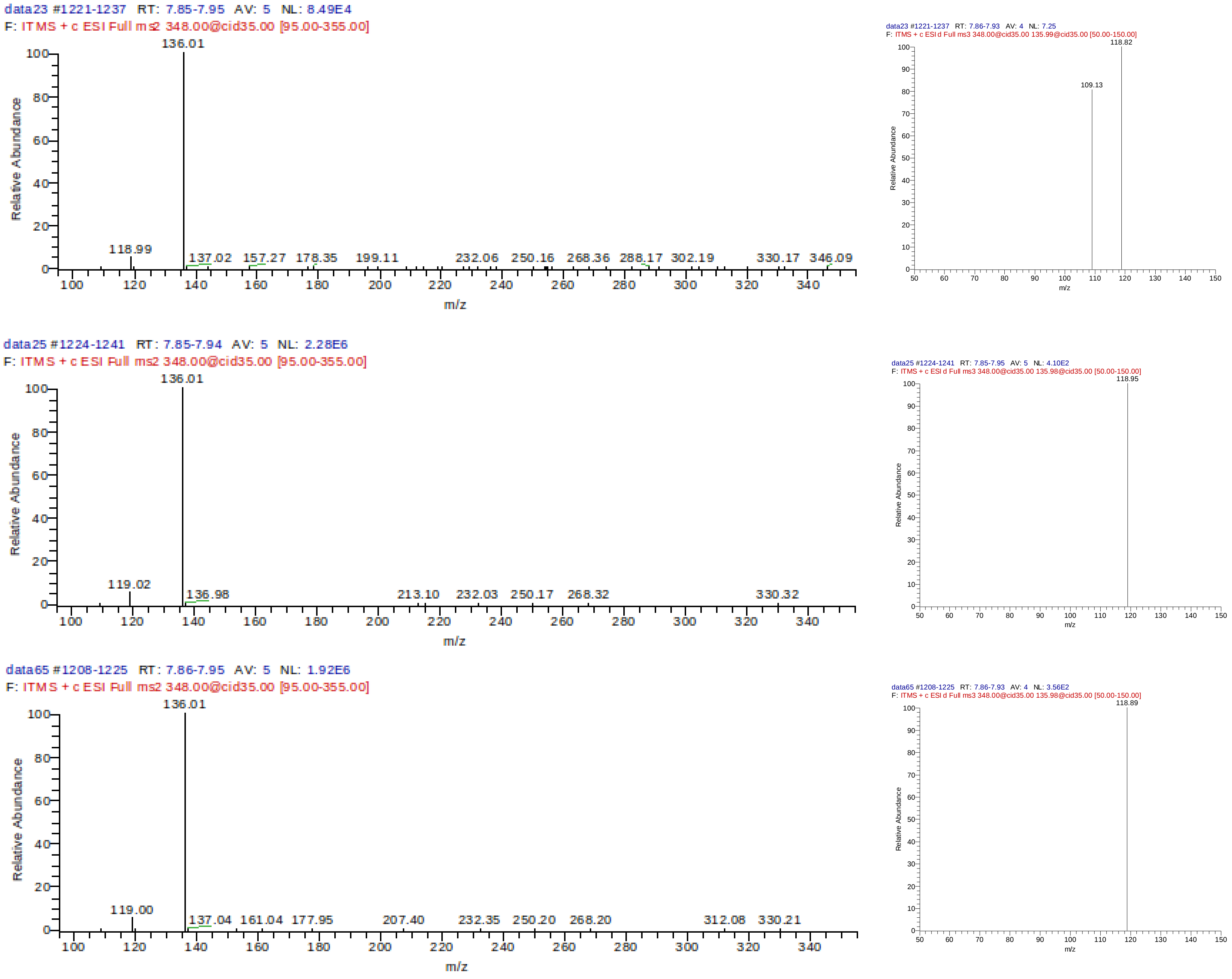

Supplementary Figure 3, ctd.

Inosine M+H[1+], m/z 269.0874

cell extract

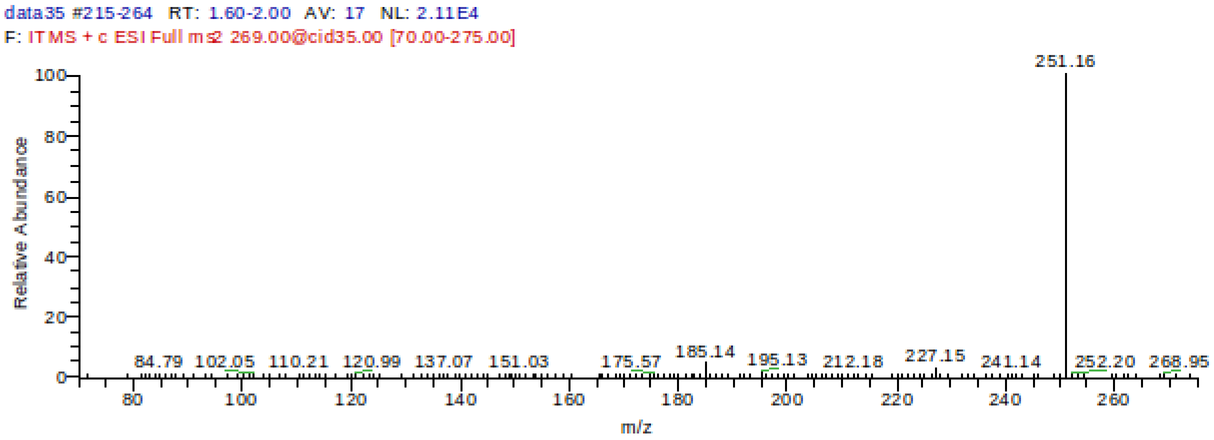

cell extract
+
chemical

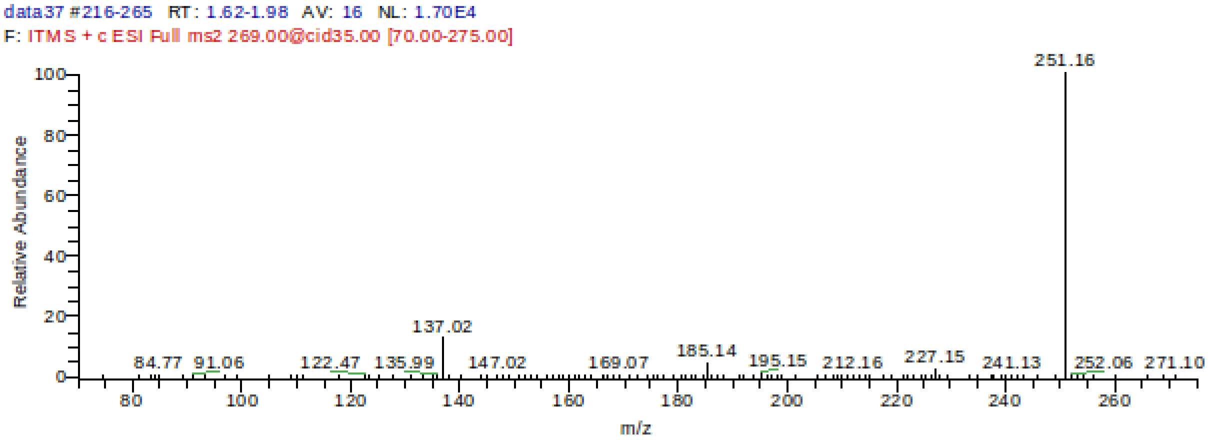

chemical
reference

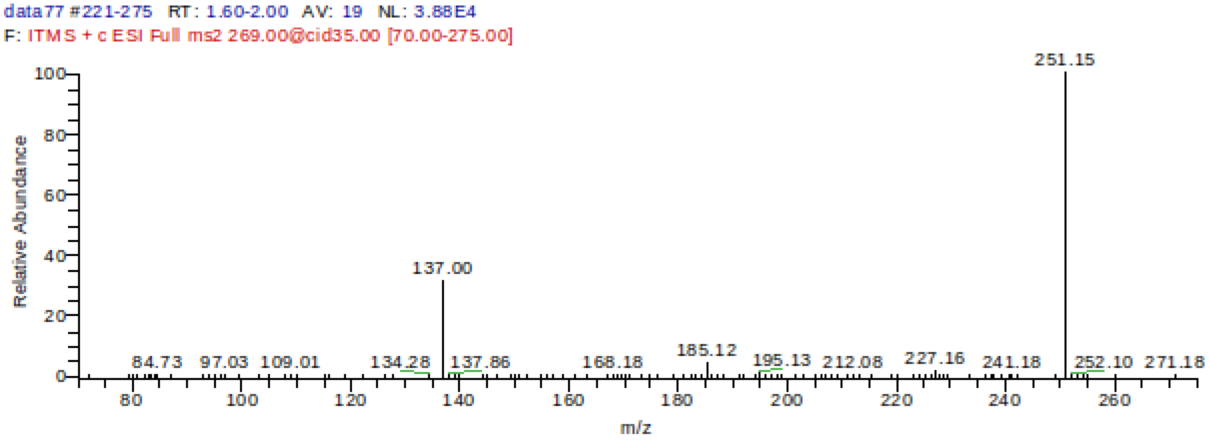

Supplementary Figure 3, ctd.

Xanthine M+H[1+], m/z 153.04

cell extract

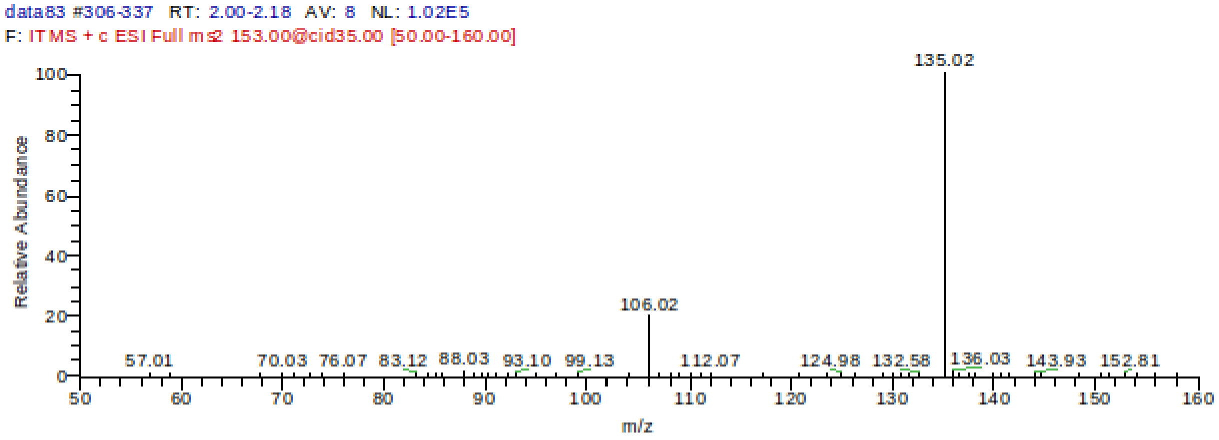

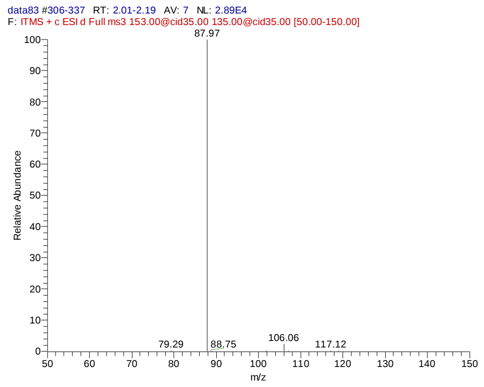

cell extract
+
chemical

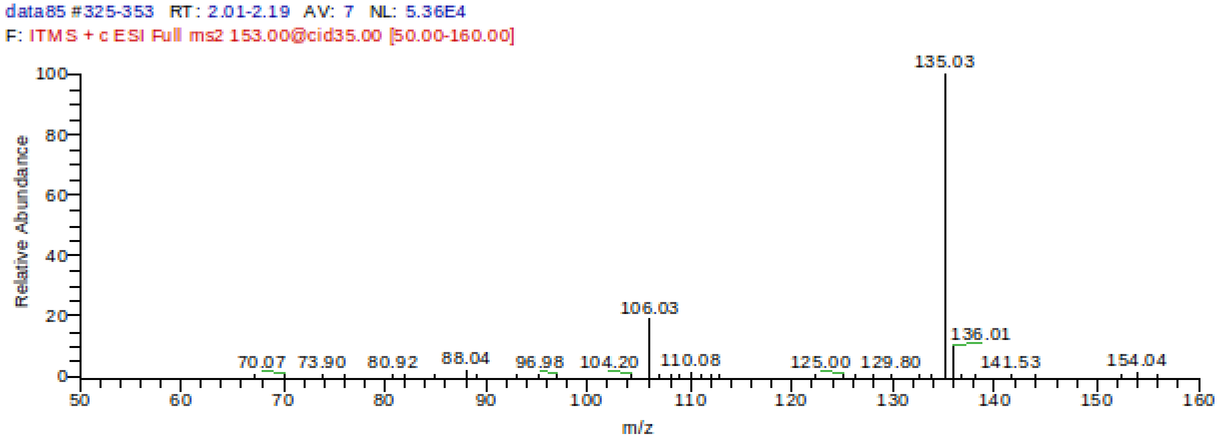

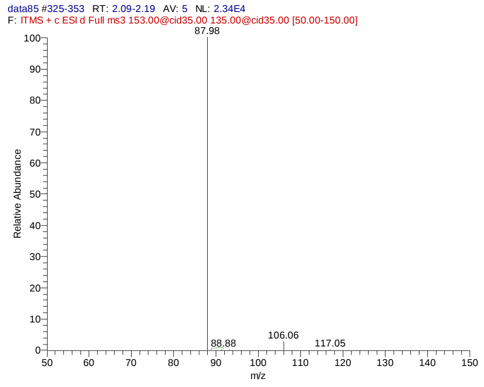

chemical
reference

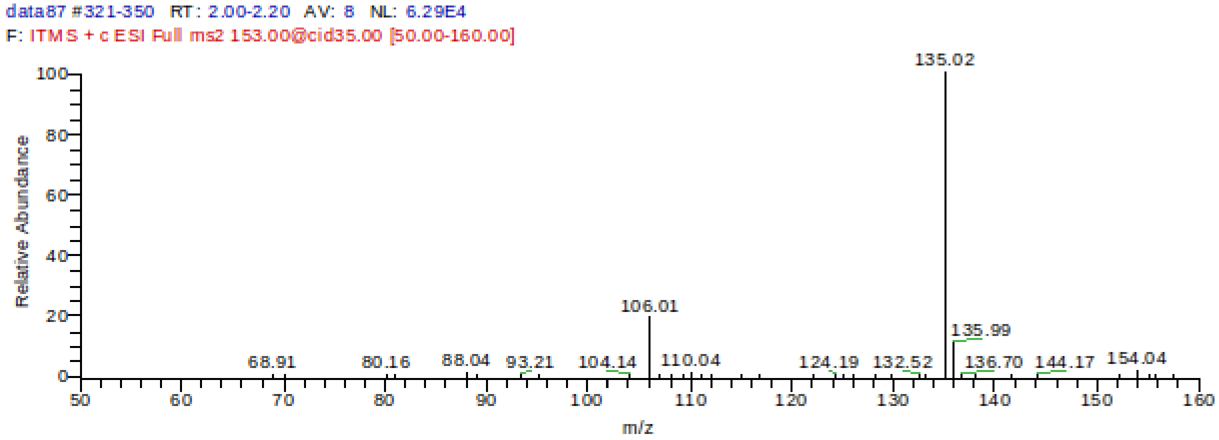

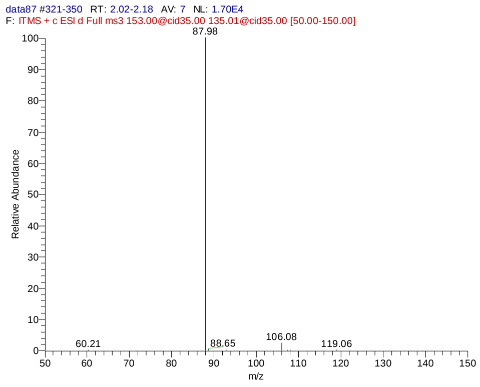

Supplementary Figure 3, ctd.

GMP M+H[1+], m/z 364.07

cell extract

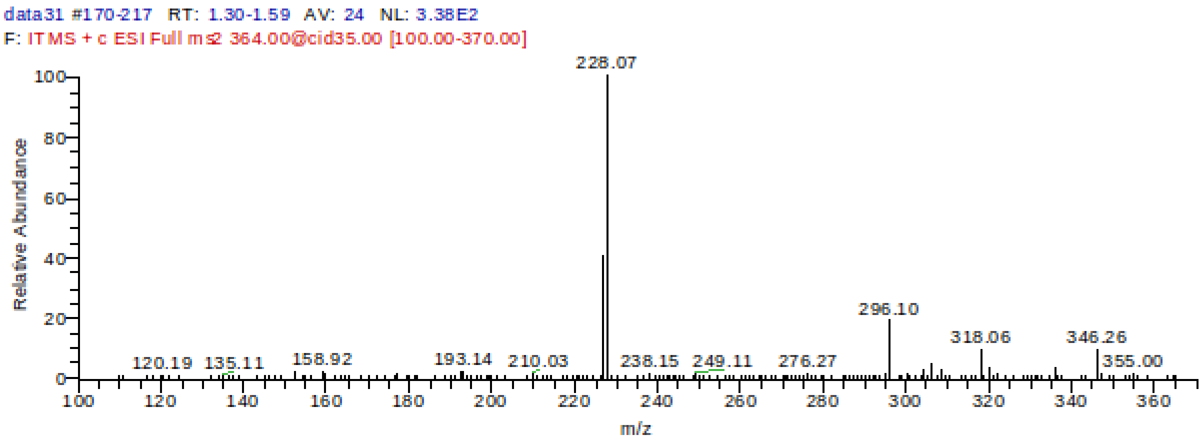

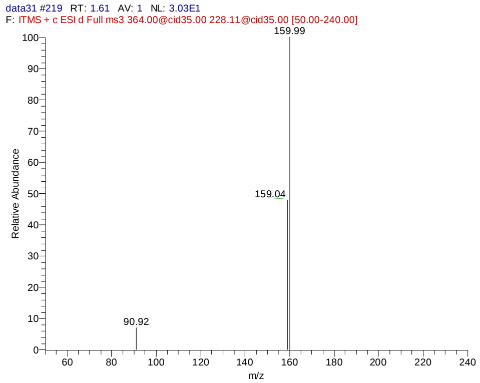

cell extract
+
chemical

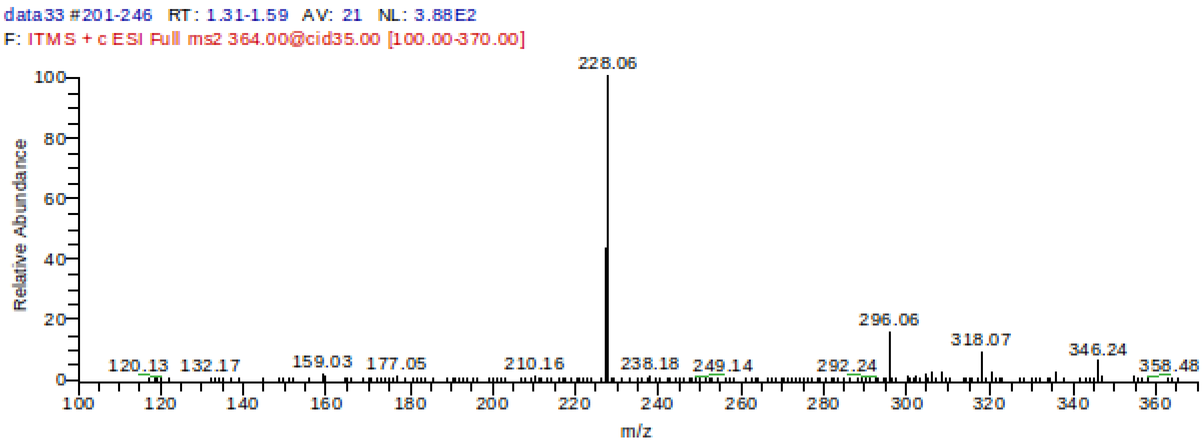

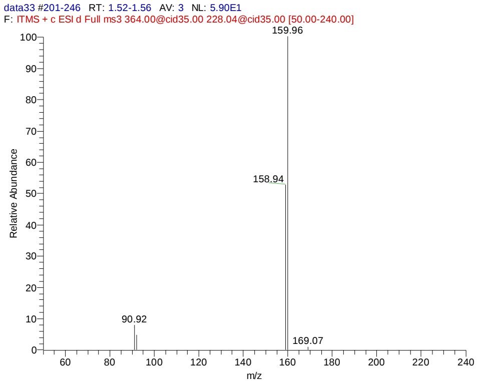

chemical
reference

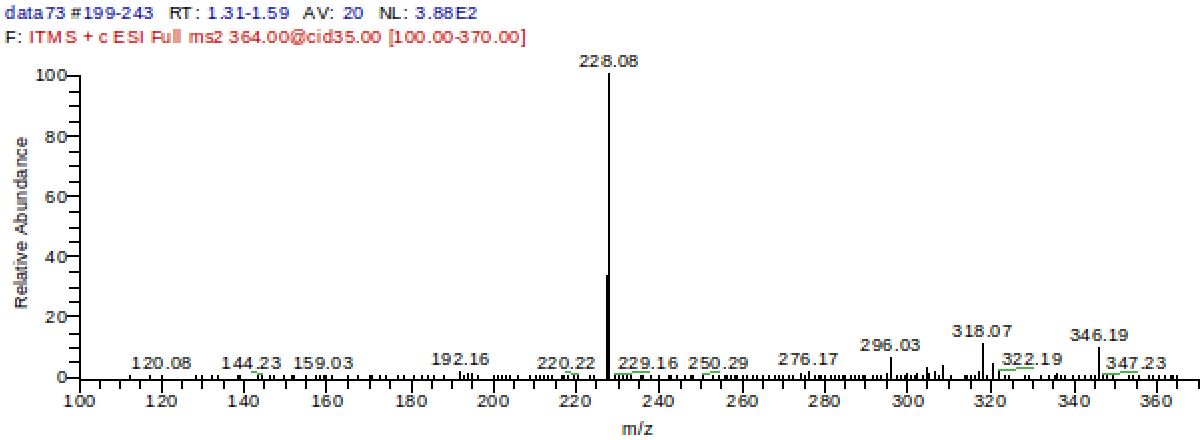

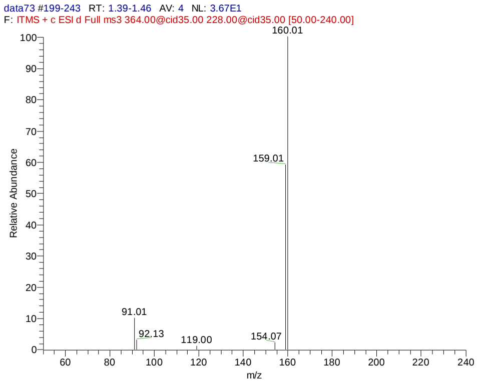

Supplementary Figure 3, ctd.

Glutamate M+H[1+], m/z 148.0601

cell extract

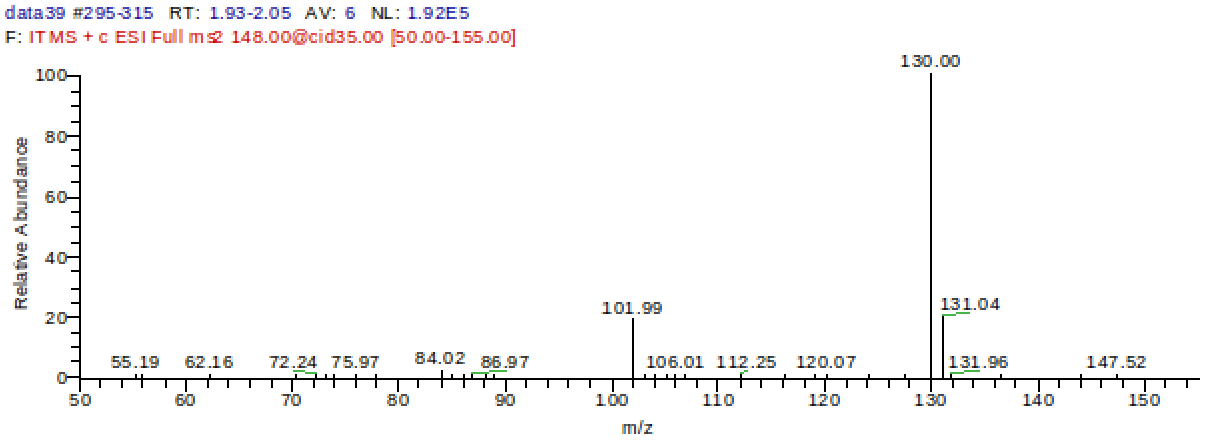

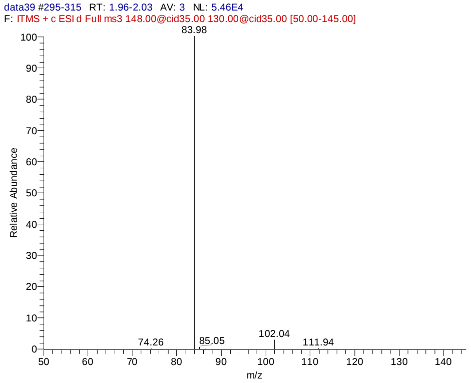

cell extract
+
chemical

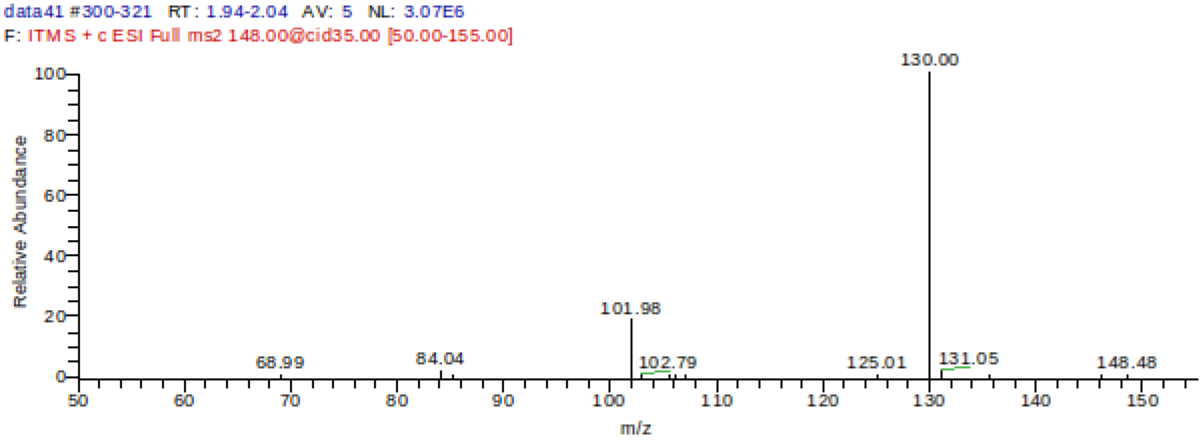

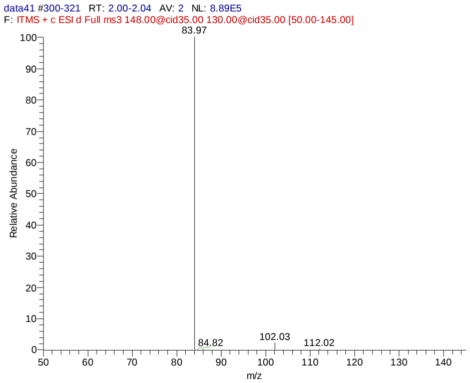

chemical
reference

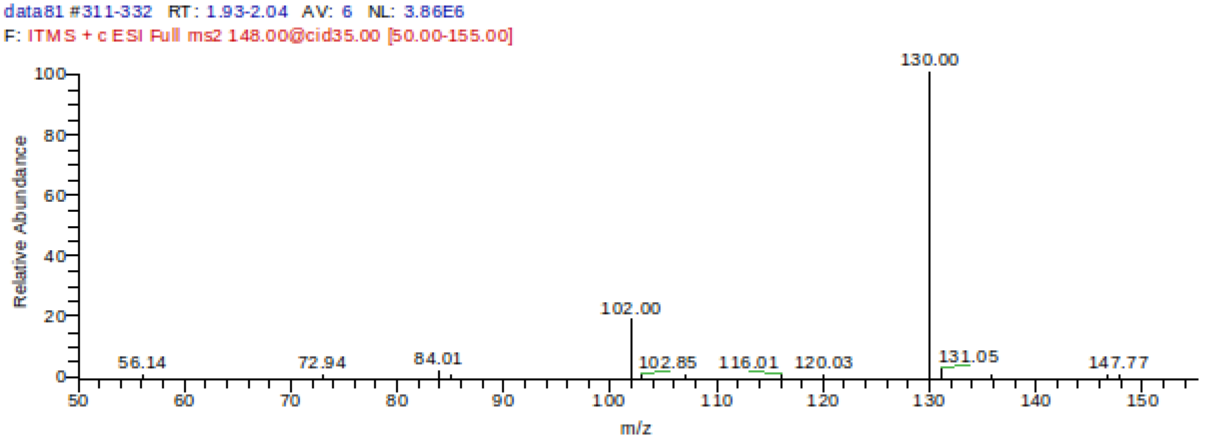

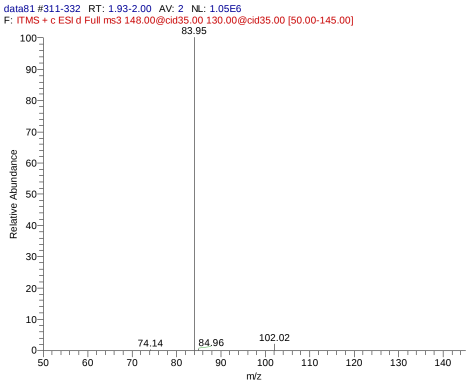

Supplementary Figure 3, ctd.

Glutathione M+H[1+], m/z 308.0896

cell extract

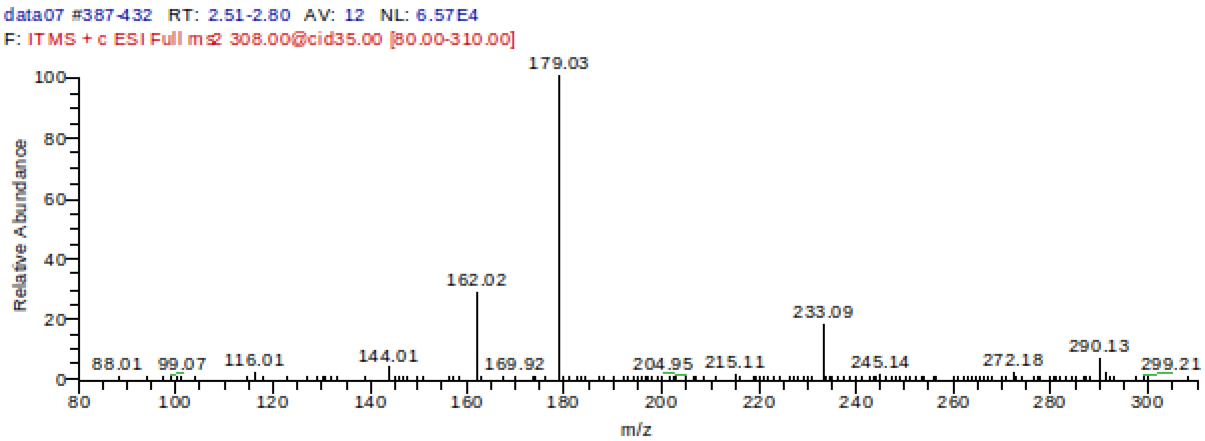

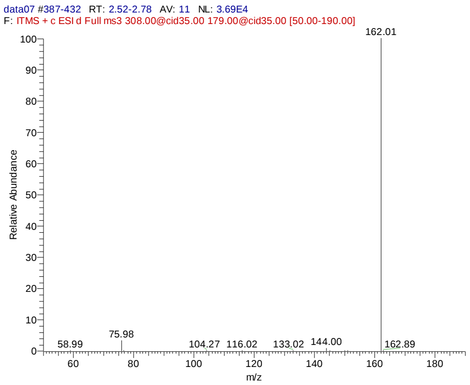

cell extract
+
chemical

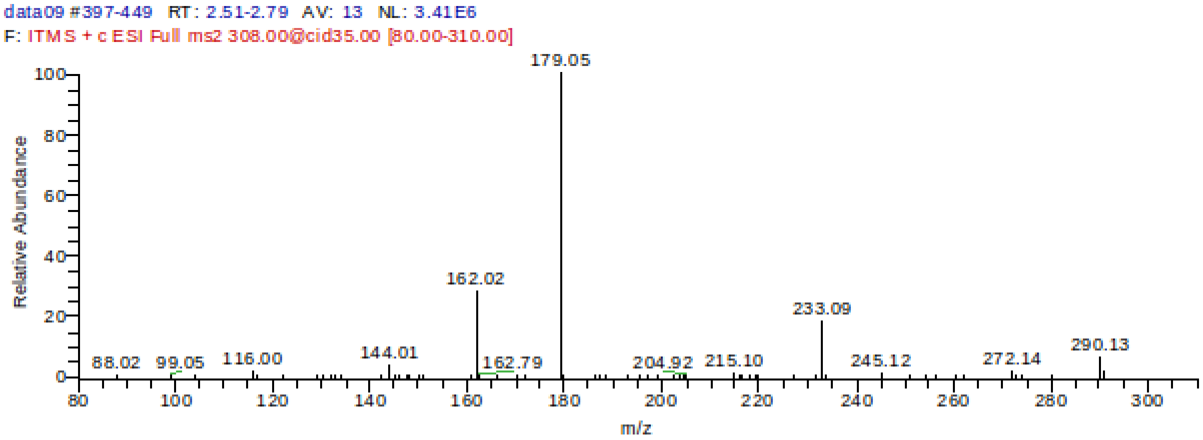

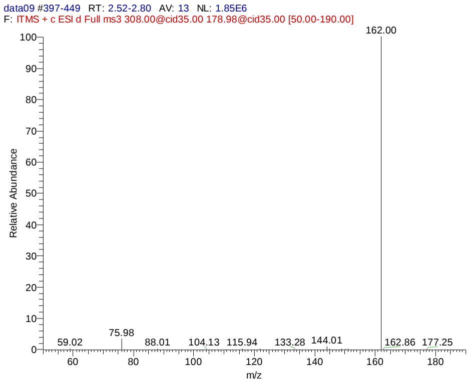

chemical
reference

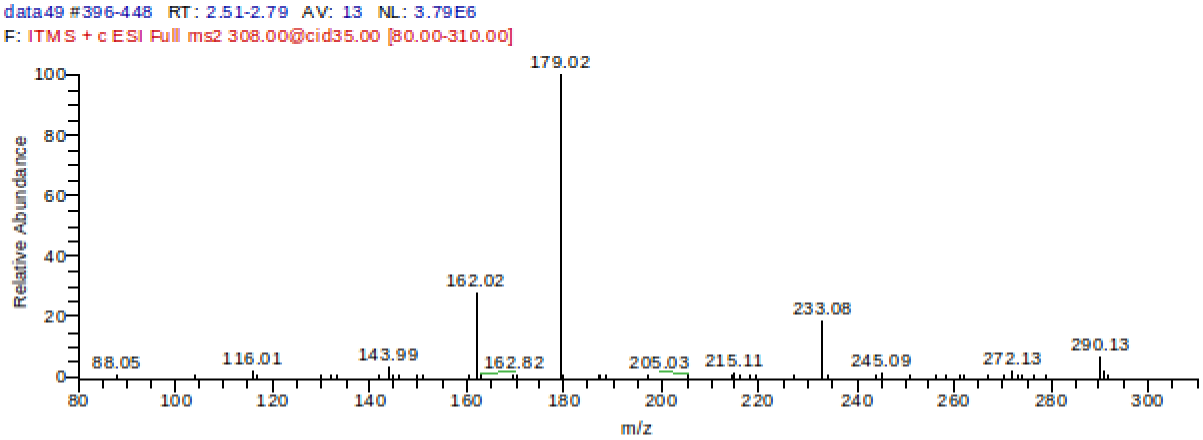

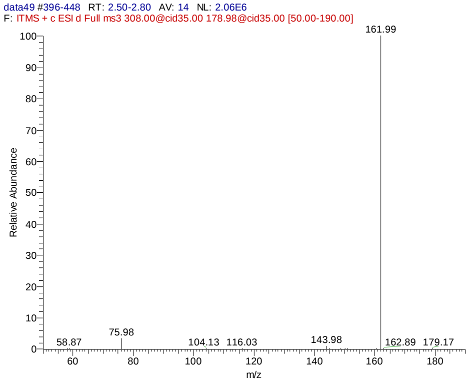

Supplementary Figure 3, ctd.

GSSG M+2H[2+], m/z 307.0829

cell extract

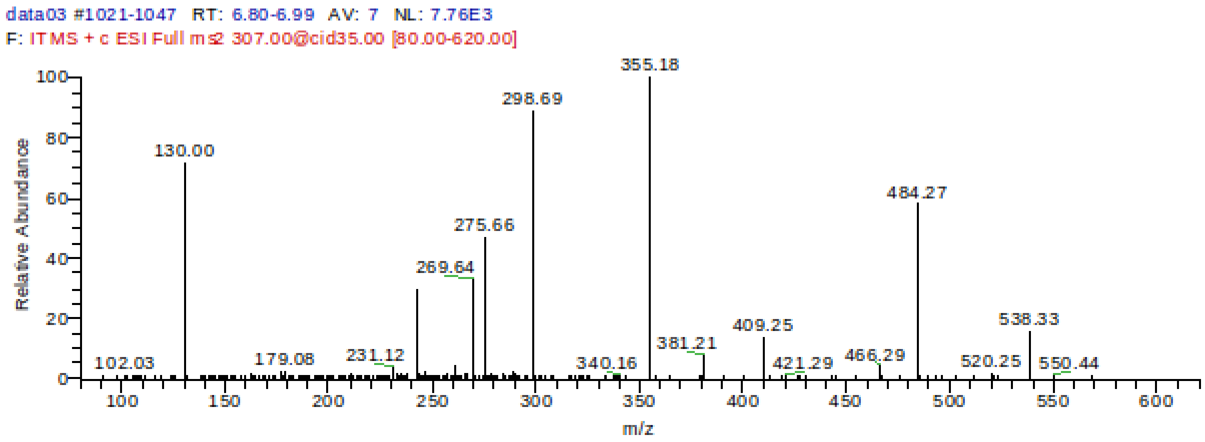

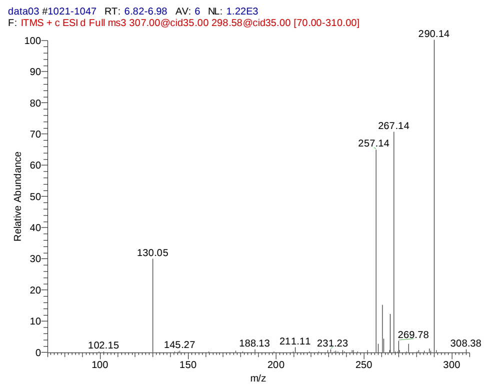

cell extract
+
chemical

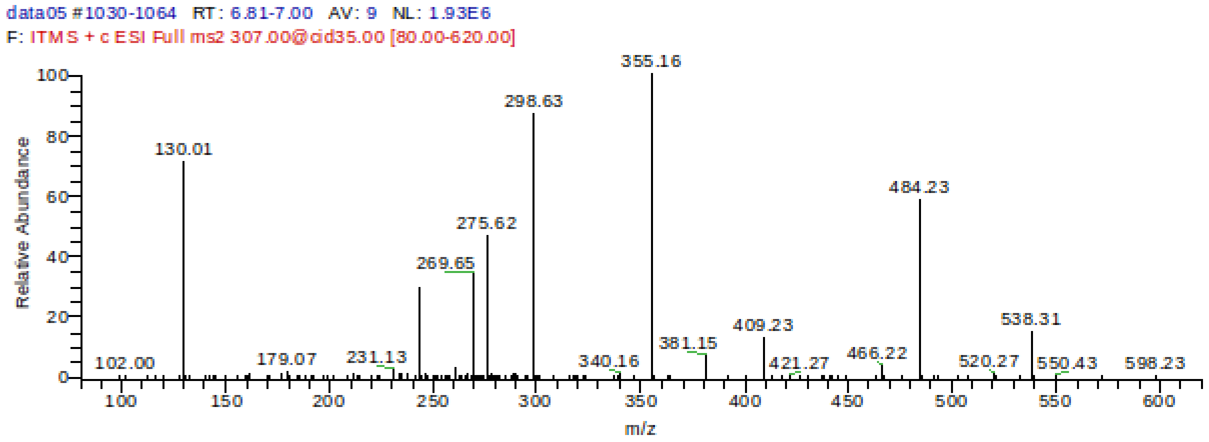

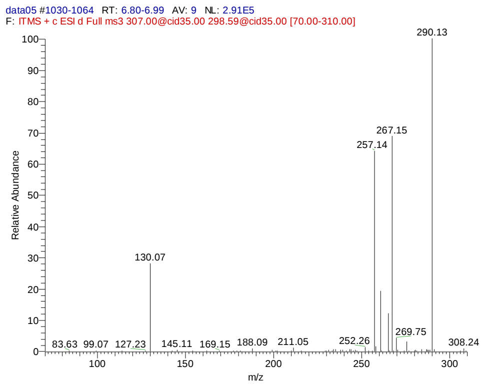

chemical
reference

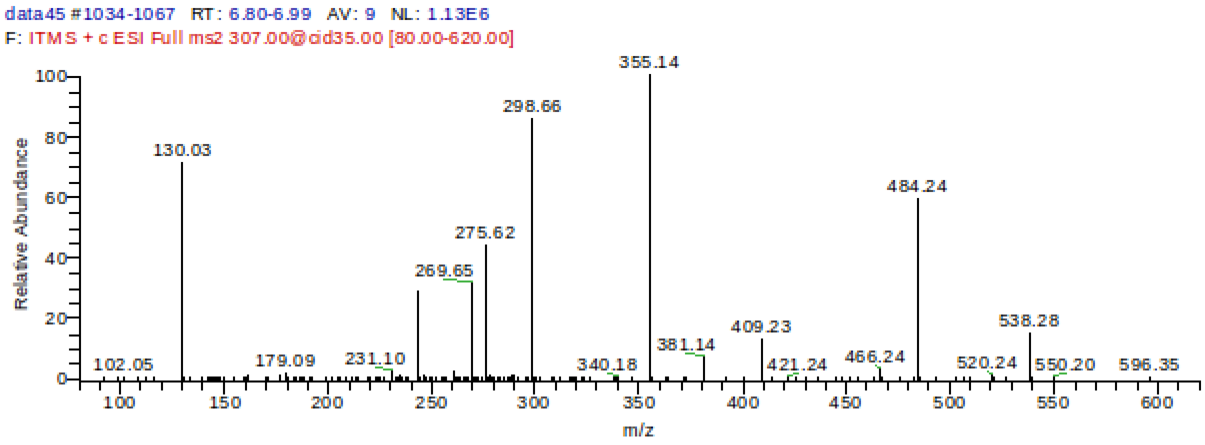

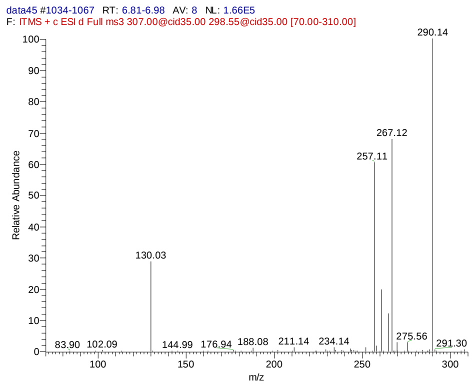

Supplementary Figure 3, ctd.
