# Supplementary material for: Predicting Network Activity from High Throughput Metabolomics
Source: PLoS Comput Biol. 2013 Jul 4;9(7):e1003123. doi: 10.1371/journal.pcbi.1003123 (PMC3701697; doi:10.1371/journal.pcbi.1003123)
Supplement: Table S1 — Pathway analysis by mummichog revealed a number of pathways activated in the infection of moDCs by yellow fever virus. Activities in many of these pathways are also identified in the significant network modules. (PDF) [file pcbi.1003123.s011.pdf]

pathway	overlap_ size	pathway_ size	enrichment p-value	adjusted p-value
Purine metabolism	21	53	0.0026	0.0020
Aspartate and asparagine metabolism	24	67	0.0063	0.0021
Aminosugars metabolism	14	33	0.0068	0.0022
Sialic acid metabolism	12	29	0.0150	0.0024
Vitamin B9 (folate) metabolism	8	17	0.0197	0.0028
Hexose phosphorylation	8	18	0.0287	0.0031
Drug metabolism - cytochrome P450	13	37	0.0475	0.0034
Vitamin B1 (thiamin) metabolism	5	10	0.0488	0.0050
Vitamin B3 (nicotinate and nicotinamide) metabolism	8	21	0.0719	0.0050
Histidine metabolism	8	21	0.0719	0.0050
Tryptophan metabolism	20	69	0.1083	0.0052
N-Glycan biosynthesis	6	14	0.0675	0.0055
Glutathione Metabolism	5	11	0.0737	0.0067
Methionine and cysteine metabolism	15	51	0.1377	0.0070
Tyrosine metabolism	23	86	0.1784	0.0084
Glycerophospholipid metabolism	13	44	0.1559	0.0085
Ubiquinone Biosynthesis	4	8	0.0779	0.0085
Glycosphingolipid biosynthesis - globoseries	4	8	0.0779	0.0085
Glutamate metabolism	5	12	0.1042	0.0091
Putative anti-Inflammatory metabolites formation from EPA	7	20	0.1330	0.0094
Pyrimidine metabolism	13	45	0.1776	0.0100
