# Supplementary material for: Predicting Network Activity from High Throughput Metabolomics
Source: PLoS Comput Biol. 2013 Jul 4;9(7):e1003123. doi: 10.1371/journal.pcbi.1003123 (PMC3701697; doi:10.1371/journal.pcbi.1003123)
Supplement: Table S2 — Primer sequences and references for quantitative PCR. (PDF) [file pcbi.1003123.s012.pdf]

Gene	Primer sequences (forward, reverse)	references
B2M	AAGATTCAGGTTTACTCACGTC<br>TGATGCTGCTTACATGTCTCG	Scheres et al. (2010) J Periodontal Res. 45(2):262-70.
IL-6	GGCACTGGCAGAAAACAACC<br>GGCAAGTCTCCTCATTGAATCC	Scheres et al. (2010) J Periodontal Res. 45(2):262-70.
iNOS	GCG ACA GAG ACA GGA AAA TC<br>GTA CTT ATC CAT GCA GAC AAC C	Yao et al. (2010) Nitric Oxide. 22(3):197-204.
nNOS	GGC AGA GAT GAA AGA TAT GGG<br>GAA TAT GGG TTG TTG AGG ACG	Yao et al. (2010) Nitric Oxide. 22(3):197-204.
eNOS	CCA GGA AAC GGT CGC TT<br>CAA ACC ACT CCA GCG T	Yao et al. (2010) Nitric Oxide. 22(3):197-204.
GCLC	GATGCTGTCTTGCAGGGAATG<br>AGCGAGCTCCGTGCTGTT	Coppin et al. (2008) JBC 283:19342–19350
GCLM	GAAGCAAGTTTCCAAGAAGCTCTT<br>CTCTACTTTTCACAATGACCGAATACC	Coppin et al. (2008) JBC 283:19342–19350
GSS	CCTGCTAGTGGATGCTGTCA<br>GGACTTGCTTGTGGATGTCA	Coppin et al. (2008) JBC 283:19342–19350
